# GamblingLess: A Randomised Trial Comparing Guided and Unguided Internet-Based Gambling Interventions

**DOI:** 10.3390/jcm10112224

**Published:** 2021-05-21

**Authors:** Nicki A. Dowling, Stephanie S. Merkouris, Simone N. Rodda, David Smith, Stephanie Aarsman, Tiffany Lavis, Dan I. Lubman, David W. Austin, John A. Cunningham, Malcolm W. Battersby, Seung Chul O

**Affiliations:** 1 School of Psychology, Deakin University, Geelong, VIC 3220, Australia; stephanie.merkouris@deakin.edu.au (S.S.M.); stephanie.aarsman@deakin.edu.au (S.A.); david.austin@deakin.edu.au (D.W.A.); 2 Melbourne Graduate School of Education, University of Melbourne, Parkville, VIC 3010, Australia; 3 School of Population Health, University of Auckland, Private Bag 92019, Auckland 1142, New Zealand; s.rodda@auckland.ac.nz; 4 Turning Point, Eastern Health, 110 Church St, Richmond, VIC 3121, Australia; 5 College of Medicine and Public Health, Flinders University, Bedford Park, SA 5042, Australia; david.smith@flinders.edu.au (D.S.); malcolm.battersby@flinders.edu.au (M.W.B.); 6 School of Psychology, Faculty of Health and Medical Sciences, The University of Adelaide, Adelaide, SA 5000, Australia; tiffany.lavis@adelaide.edu.au; 7 Turning Point, Eastern Health and Monash Addiction Research Centre, Eastern Health Clinical School, Richmond, VIC 3121, Australia; dan.lubman@monash.edu; 8 National Addiction Centre, Institute of Psychiatry, Psychology and Neuroscience, King’s College London, London WC2R 2LS, UK; john.cunningham@camh.ca; 9 Centre for Addiction and Mental Health, Toronto, ON M6J 1H4, Canada; 10 Department of Psychiatry, University of Toronto, Toronto, ON M5T 1R8, Canada; 11 Faculty of Health, Deakin University, Geelong, VIC 3220, Australia; eric.o@deakin.edu.au

**Keywords:** gambling, internet, online, intervention, treatment, cognitive-behavioural, CBT, self-help, self-directed, guidance, guided, unguided

## Abstract

There is little evidence relating to the effects of adding guidance to internet-based gambling interventions. The primary aim was to compare the effectiveness of an online self-directed cognitive-behavioural gambling program (GamblingLess) with and without therapist-delivered guidance. It was hypothesised that, compared to the unguided intervention, the guided intervention would result in superior improvements in gambling symptom severity, urges, frequency, expenditure, psychological distress, quality of life and help-seeking. A two-arm, parallel-group, randomised trial with pragmatic features and three post-baseline evaluations (8 weeks, 12 weeks, 24 months) was conducted with 206 gamblers (106 unguided; 101 guided). Participants in both conditions reported significant improvements in gambling symptom severity, urges, frequency, expenditure, and psychological distress across the evaluation period, even after using intention-to-treat analyses and controlling for other low- and high-intensity help-seeking, as well as clinically significant changes in gambling symptom severity (69% recovered/improved). The guided intervention resulted in additional improvements to urges and frequency, within-group change in quality of life, and somewhat higher rates of clinically significant change (77% cf. 61%). These findings, which support the delivery of this intervention, suggest that guidance may offer some advantages but further research is required to establish when and for whom human support adds value.

## 1. Introduction

Cognitive-behavioural therapy (CBT) and motivational interviewing (MI) are considered to be “best-practice” psychological interventions for problem gambling [1,2,3,4]. Only a small proportion of people with gambling problems, however, access gambling treatment services, which are typically offered face to face [5], suggesting that this delivery model does not provide adequate access to evidence-based treatment [6]. Barriers to treatment, which are now well documented, include personal factors (e.g., shame, denial, stigma, and a desire to self-manage), as well as resource limitations (e.g., limited availability of trained clinicians, treatment costs, and childcare requirements), geographic inaccessibility, and time constraints [7]. Self-directed interventions are increasingly becoming employed to overcome these barriers [8]. These interventions have extended support for health behaviour change beyond standard treatment contexts by having the potential to support ‘hard to reach’ or underserved populations, such as those who are unable or unwilling to participate in other interventions [9,10,11].

### 1.1. Internet-Based Interventions

Advances in technology have resulted in an increased focus on self-directed material being delivered via the internet. Following an evidence-based classification of internet-supported interventions, Barak, Klein, and Proudfoot [12] described internet (web)-based therapeutic interventions as prescriptive online programs operated through websites that aim to create positive cognitive, behavioural, and emotional change. These are highly structured programs with comprehensive content presented in a modularised format. The content of these interventions, which is frequently modelled on effective face-to-face treatment programs, is informed by intervention theory, such as CBT (iCBT). These interventions have many advantages, including high accessibility and availability, anonymity, convenience, portability, cost-effectiveness, and low burden, as well as the potential for scalability, real-world translation, and accurate data recording [9,10,11]. There is considerable meta-analytic evidence that such interventions are effective in the treatment of psychiatric and addictive disorders and can produce equivalent overall effects as face-to-face treatment [13,14,15].

Internet-based therapeutic interventions can be automated interventions, independent of human support (self-guided, unguided, or pure self-directed [PSD] interventions), or can involve minimal human support (guided self-directed [GSD] interventions) [6,16]. GSD interventions are generally characterised by supportive or facilitative guidance, with the aim of actively guiding clients in the use of a self-directed protocol [17,18]. Guides provide motivational support, monitor progress, clarify information, review activities and address any technical questions, rather than delivering therapeutic content [17,19,20]. Meta-analytic evidence suggests that GSD interventions for psychiatric and addictive disorders are more effective than control groups [14,21] and PSD interventions [14,22], and can be as effective as more intensive face-to-face therapies [18,23,24,25]. They also appear to be acceptable to diverse clients in different treatment settings across multiple countries [6,26,27] and there are encouraging results for their cost-effectiveness [28].

### 1.2. Internet-Based Gambling Interventions

Despite evidence of their efficacy in other fields, there is a paucity of research investigating the effectiveness of internet-based therapeutic interventions for the treatment of problem gambling. A limited number of recent randomised trials have, however, found that PSD iCBT for problem gambling is effective in improving gambling and psychological outcomes up to 12 months following treatment [29,30,31,32,33,34], although some have found no differences compared to a no intervention control group [31] or brief online personalised feedback [34]. Moreover, adding a separate mental health [33] or alcohol use intervention [32] to iCBT has not improved outcomes.

Several studies have also evaluated these interventions with minimal therapist contact, mostly comprising weekly 15–45 min telephone calls or email counselling. Several uncontrolled trials have found that iCBT with therapist support resulted in improvements across gambling and psychological outcomes up to 36 months following treatment [35,36,37,38]. These findings are supported by a randomised controlled trial (RCT) in which iCBT with minimal therapist contact resulted in significant post-treatment improvements in gambling and psychological outcomes compared to a waitlist control group that were maintained up to 36 months post-treatment [39]. There is also evidence that therapist-supported iCBT is comparable to internet-based behavioural couples therapy in improving gambling and psychological outcomes over a 12 month follow-up period [40,41]. A recent systematic review [8] revealed that self-directed interventions with therapist contact showed a non-statistically significant advantage over those without therapist support, possibly due to the small number of studies including therapist contact.

Although this growing area of research provides promising evidence for internet-based therapeutic gambling interventions, with and without therapist support, there remains little information about the effects of adding guidance to these interventions. In 2016, a study conducted by Luquiens et al. [42] found that following screening of commercial online poker site consumers, there were no significant differences in problem gambling severity or gambling expenditure outcomes across three conditions (personalised normative feedback email; downloadable CBT self-help workbook; workbook plus six sessions of personalised therapist-delivered email guidance). Moreover, the guidance group displayed no significant improvements over time and displayed the highest rates of attrition. The authors concluded that guidance may have adverse effects in internet-based interventions for gamblers who do not seek help, possibly because it is too time consuming or too intrusive, or because it requires a commitment to someone they have not chosen. This study also employed a downloadable CBT self-help workbook in PDF format, rather than an internet-based therapeutic intervention, as described by Barak et al. [12]. The influence of guidance in internet-based therapeutic interventions for gamblers who seek treatment therefore remains unresolved. 

### 1.3. Characteristics of Gamblers Accessing Internet-Based Interventions

There is now some evidence that the characteristics of gamblers who use internet-based therapeutic interventions resemble other treatment-seeking populations in terms of demographic characteristics and the severity of the problem [34]. The extraction of a profile of participants accessing internet-based therapeutic gambling interventions from the available studies reveals that overall, participants are mostly men aged between 32 and 48 years who are educated and employed. They are characterised by problematic gambling on electronic gaming machines (EGMs), gambling severity classified in the problem gambling category (81–96%), and high rates of mental health and substance use difficulties. Approximately one-third to one-half report previous gambling treatment. This profile is very similar to that reported by gamblers using the Australian national online gambling service [43,44].

### 1.4. Factors Associated with Internet-Based Gambling Intervention Outcomes

Much less is known about the factors associated with the outcomes associated with internet-based gambling interventions. There is some evidence that better outcomes are associated with factors such as younger age, lower pre-treatment gambling severity, lower levels of dissociation while gambling, debts due to gambling, lack of previous treatment, lower alcohol consumption, higher pre-treatment self-efficacy, and treatment engagement [29,30,34,35]. Better treatment outcomes are also predicted by greater improvement on cognitive distortions, some indices of psychopathology, and psychiatric distress [38]. Moderators of improvements in problem gambling severity include low vocational status, higher baseline loneliness, and less severe post-treatment gambling-related symptoms [29]. More generally, systematic review evidence [45] suggests that male sex and low depression levels were the most consistent predictors of successful treatment outcomes across multiple time points for psychological gambling treatments. Likely predictors of successful treatment outcomes also included older age, lower gambling symptom severity, lower levels of gambling behaviours, and lower levels of alcohol use. At a minimum of one time point, significant associations were identified between successful treatment outcomes and being employed, ethnicity, and readiness to change. Mixed results were identified for treatment goal and there were an insufficient number of studies examining quality of life to draw valid conclusions. Income, preferred gambling activity, anxiety, psychological distress, substance use, prior gambling treatment and medication use were not significantly associated with treatment outcomes at any time point.

The factors associated with engagement and attrition to internet-based gambling interventions have yet to be examined, although there are few clear predictors of these factors in other literatures [46,47]. For example, systematic evidence suggests [46] that the results from the literature examining the predictors of treatment adherence to online psychological interventions, defined as the amount of a therapeutic intervention that an individual engages with or completes, are either too preliminary or are mixed. This literature suggests that female gender and computer factors (literacy and technical difficulties) consistently predict higher adherence. Age and readiness to change have demonstrated mixed findings, alcohol use and self-efficacy have demonstrated inconclusive findings with few too studies from which to draw conclusions, and the majority of other variables (such as employment, ethnicity, and symptom severity) do not predict adherence. Similarly, a systematic review by Melville et al. [47] found that several factors, including older age, gambling frequency, comorbid anxiety, and comorbid drug or alcohol use disorders have been positively associated with psychological gambling treatment dropout but that findings were mixed; that sex, income, gambling expenditure, problem gambling severity, type of gambling, and comorbid depression have been consistently not associated with treatment dropout; and that factors such as ethnicity, motivation to stop gambling, gambling urges, self-efficacy, prior treatment experience and treatment motivation have not been associated with dropout but there are few too studies from which to draw conclusions. An enhanced understanding of the factors associated with successful treatment outcomes will allow for the identification of subgroups of gamblers who are most likely to respond to interventions delivered via internet technologies [45,47].

### 1.5. Aims and Hypotheses

A growing literature generally supports the use of internet-based interventions for problem gambling, but it remains uncertain as to whether GSD interventions offer any advantages over PSD interventions. The primary aim of this study was therefore to compare the effectiveness of GamblingLess, a high-intensity online self-directed cognitive-behavioural program for gambling, delivered under PSD conditions (without any practitioner guidance) and GSD conditions (with guidance provided via email by practitioners from existing gambling treatment services). A two-arm, parallel-group, randomised trial with pragmatic trial features was conducted to compare these two conditions at 8 week, 12 week, and 24 month post-baseline evaluations. It was hypothesised that the GSD intervention would lead to better outcomes than the PSD intervention, as assessed by reductions in gambling symptom severity (primary outcome measure), gambling urges, gambling behaviours (frequency, expenditure) and psychological distress; and increases in quality of life and help-seeking (low intensity, high intensity). Secondary aims were to explore the profile of GamblingLess users; and explore the predictors of treatment outcomes, engagement, and post-baseline evaluation completion. The experiences and perceptions of the practitioners supporting the GSD condition has been published separately [48].

## 2. Methods

### 2.1. Trial Design

Initially, a two-arm, parallel-group, randomised trial with pragmatic features and online evaluations at 8 and 12 weeks from the pre-intervention (baseline) assessment was conducted, with a view to conducting a longer-term evaluation [12 months post-baseline in the trial protocol: 49]. Additional funding was subsequently obtained to conduct this evaluation at 24 months from the pre-intervention assessment. The trial was registered with the Australian New Zealand Clinical Trials Registry (Trial ID: ACTRN12615000864527) and the full trial protocol is published elsewhere [49]. The trial closely adhered to the protocol and detailed information about the GamblingLess program and trial methodology can be found in the trial protocol [49]. This study was approved by the Deakin University Human Research Ethics Committee (Ethics ID: 2014-123) and the Eastern Health Human Research Ethics Committee (Ethics ID: E07/2015).

### 2.2. Participant Recruitment and Registration

Participants were recruited Australia-wide via advertisements with links to the GamblingLess website in: public places and health services (e.g., general practitioner [GP] waiting rooms), Facebook and Google (Google Adwords), and various university and gambling-related websites (e.g., Gambling Help Online, Victorian Responsible Gambling Foundation). Participants were also recruited via counsellors from current gambling treatment services, including the participating agencies. Individuals were eligible to participate if they: (1) resided in Australia; (2) expressed interest in seeking help for their own gambling problems; (3) were 18 years of age or older; (4) had access to the internet; (5) had adequate knowledge of the English language; and (6) were willing to take part in the program and complete brief assessment measures at registration and follow up. Consistent with typical pragmatic trials, the program was available to individuals who were seeking other forms of assistance [50]. Participants registered for the program by providing an email address and password, confirming their eligibility by indicating that they were 18 years of age or older and that they lived in Australia, and providing informed consent online. Upon providing consent, participants were immediately directed to the online pre-intervention questionnaire, after which they were automatically randomised to one of the two intervention conditions. 

### 2.3. Interventions

#### 2.3.1. The GamblingLess Program

The GamblingLess program is an internet-based cognitive-behavioural therapeutic intervention designed to help people with gambling problems. The program was developed as a comprehensive and intensive cognitive-behavioural program that emulates the intensity and depth of a face-to-face cognitive-behavioural intervention, and from which more brief and targeted online and mobile self-directed interventions can be developed. This program comprises four modules, each ranging from 13 to 15 activities and designed to take approximately one to two hours to complete. The program comprises motivational interviewing, behavioural, cognitive, and relapse prevention modules (see [49] for a detailed description of the program).

The GamblingLess program was designed as an 8 week intervention, consistent with the completion of one module per fortnight. While it was recommended that participants complete all modules and activities in numerical order, they were allowed to complete as many activities as they liked in any order they chose. It was therefore not anticipated that participants would complete all activities in each module. Participants in both conditions were encouraged to complete the GamblingLess program across an 8 week period but were given access to the program for 14 weeks post-baseline, consistent with a pragmatic trial of real-world online interventions [51] but with a clear deadline provided for treatment duration to enhance compliance [17,52]. 

#### 2.3.2. Guidance in the GSD condition 

Participants in the GSD condition were provided with weekly appointment-based email guidance [20] across an 8 week period, consistent with the completion of one module per fortnight. Guidance consisted of a maximum of one contact per week with a maximum duration of 20 min per contact, as per recommendations [18]. Guides were responsible for providing assistance in a supportive and facilitative manner, with the aim of orienting participants in the use of the GamblingLess program [17,18]. Consistent with the GSD literature [17,19,20], the guides provided support, monitored progress, clarified information contained within the program, answered technical questions, addressed other problems that arose, and provided reminders to complete modules. 

Eleven guides were recruited from current gambling treatment services in Australia, namely, the Australian national online counselling service (Gambling Help Online) and the Victorian face-to-face gambling counselling services (Gamblers Help). All emails exchanged between the guides and their allocated participants were via secure project-specific email addresses. The guidance component of the GSD intervention was manualised and the guides were required to complete a 3 h workshop in which they were trained in the use of the GamblingLess program, how to communicate via email and how to provide guidance via email (SM and SR). A group peer supervision session moderated by a member of the research team (SM), which was conducted part way through this study, included a discussion on the content of the e-mail correspondence, sharing of the experiences of providing guidance, and discussions about problems encountered. In addition, the guides were provided with ongoing assistance and support from members of the research team across the course of this study, as required.

### 2.4. Data Collection

Participants completed online evaluations prior to accessing the intervention and at 8 weeks, 12 weeks, and 24 months following completion of the pre-intervention evaluation. There was no attempt to maintain contact with the participants across the 24 month evaluation period. Participants were contacted via email to complete the 8 week and 12 week post-baseline evaluations online and were compensated with an AUS$30 e-gift voucher following the completion of each of these evaluations. For the 24 month evaluation, participants were initially contacted via email to organise a time to complete the evaluation via telephone and were compensated with an AUS$50 e-gift voucher following the completion of this evaluation. Participants, however, were also provided with the option of completing the 24 month evaluation online via Qualtrics. Participants who did not complete a post-baseline evaluation received two reminder emails, followed by a reminder telephone call. One participant completed the 8 week evaluation and 20 participants completed the 24 month evaluation over the telephone with the assistance of a research assistant who was blind to the participant’s treatment condition. Given the nature of the intervention, neither participants nor guides could be blinded. Further information about the measures is provided in the trial protocol [49].

#### 2.4.1. Outcome Measures

The primary outcome was the 12-item Gambling Symptom Assessment Scale (G-SAS) [53], which was designed specifically to assess changes in gambling symptom severity during treatment. Total G-SAS scores range from 0 to 48, which can be categorised as extreme (39–46), severe (29–38), moderate (19–28), or mild (8–18) past-week symptom severity. Secondary outcomes included gambling urges, gambling behaviours, psychological distress, quality of life, and additional help-seeking behaviour. Gambling urges were measured using the gambling urge subscale of the G-SAS [53], which consists of the first four items of the G-SAS, with total subscale scores ranging from 0 to 16. Past-month gambling behaviour was assessed using self-report items relating to the number of days gambled (frequency) and amount lost (expenditure) on six gambling activities: electronic gaming machines (EGMs), table games, horse/harness/greyhound racing, sports and events betting, number games (such as lotteries and bingo), and informal private games. Past-month psychological distress was measured using the 6-item Kessler 6 Psychological Distress Scale (K6) [54], in which response options range from (1) none of the time to (5) all of the time. Using the scoring based on Australian norms, item scores are summed to obtain a total score between 6 and 30 and respondents can be classified as being at low (score of 6–13), moderate (score of 14–18), high (score of 19–24), or very high (score of 25–30) risk. The first item from the EUROHIS-QOL index was employed to assess overall quality of life: ‘How would you rate your quality of life?’ [55], in which the responses range from (1) very poor to (5) very good. This item is highly correlated with overall EUROHIS-QOL scores [55,56]. Finally, the Help-Seeking Questionnaire [57] was employed to identify how many times during the previous 30 days, participants had accessed high-intensity interventions (5 items, e.g., gambling counselling face to face) and low-intensity interventions (3 items, e.g., gambling helpline). 

#### 2.4.2. Descriptive and Diagnostic Measures 

Several measures were employed to explore the profile of GamblingLess users (Aim 1) and identify the predictors of treatment outcomes, treatment engagement and post-baseline evaluation completion (Aim 3). These included measures that were employed for diagnostic and sample descriptive purposes [49]. Socio-demographic characteristics included sex, age, country of birth, employment status and personal net income per year. Other descriptive and diagnostic measures included frequency of internet use, problem gambling severity, treatment goal, problem gambling activity, self-directed actions, internet use, alcohol use, and substance use. Frequency of internet use was assessed using a single item indicating how many hours participants used the internet for work/personal/education/ recreation purposes in a regular week. Past-year problem gambling severity was measured using the 9-item Problem Gambling Severity Index (PGSI) [58]. PGSI items are rated on a 4-point scale, ranging from (0) never to (3) almost always, with scores ranging from 0 to 27 that can be categorised into non-problem gambling (score of 0), low-risk gambling (scores of 1 or 2), moderate-risk gambling (scores between 3 and 7), or problem gambling (scores of 8 or higher). Treatment goal was measured using a single item, with the following response options: (i) quit (or stay quit) gambling altogether; (ii) quit (or stay quit) the gambling activities I think I have an issue with; or (iii) cut back (or stay cut back) the gambling activities I think I have an issue with. Problem gambling activity was identified on one or more of six gambling activities: EGMs, table games, horse/harness/greyhound racing, sports and events betting, number games (such as lotteries and bingo), and informal private games. Self-directed actions were measured using the 6-item Self-Directed Actions subscale of the Help-Seeking Questionnaire [57], which identifies the frequency of self-directed actions during the previous 30 days (e.g., online gambling forums). Alcohol use was measured using the Alcohol Use Disorders Identification Test-3 (AUDIT-3) [59], which measures the frequency of consuming six or more drinks on one occasion, with response options ranging from 0 (never) to 4 (daily or almost daily). Substance use was measured using a single item that identifies the frequency of illegal drug use or use of prescription medications for non-medical purposes in the previous 30 days [60].

#### 2.4.3. Process Measures 

Several brief measures were also employed to explore the processes or mechanisms that are hypothesised to be responsible for changes in gambling outcomes following the GamblingLess program [49]. In these analyses, only process measures in which entire scales were employed were included to inform Aims 1 and 3. Readiness rulers [61] were used to assess the importance (how important is it for you that you limit/stop your gambling?), readiness (where does limiting/stopping gambling fit on your list of priorities?), and confidence (how confident are you that you could resist an urge to gamble?) of participants on a scale from 1 to 10, where higher scores indicate greater importance, readiness, or confidence. Confidence in ability to resist gambling when faced with high-risk situations was measured using the Brief Situational Confidence Questionnaire (BSCQ) [62], which was adapted for gambling by adding two additional items (financial pressures, alcohol or drug use) and used modified response options from (0) not at all confident to (10) totally confident.

### 2.5. Randomisation

Participants were randomly allocated (via a random number generator) to treatment condition using a combination of “stratification group” and “block randomisation”. Participants were automatically allocated to a stratification group based on sex, median age and problem gambling severity (using PGSI scores of 8+), forming eight stratification groups. Participants were then automatically allocated to one of four sequences using block randomisation. The randomisation structure was built into the GamblingLess eResearch platform by the Deakin eMental Health Unit team. Although one member of the research team intermittently monitored the automated randomisation process to ensure no technical issues ensued, this researcher was not able to influence the randomisation sequence and had no knowledge of the forthcoming allocations.

### 2.6. Statistical Analyses

Statistical analyses were conducted using Stata 15 [63]. Descriptive statistics were provided for baseline outcome, descriptive, and process measures. Nearly half of participants (48.5%) completed the 8 week, 12 week, or 24 month evaluation. Due to these modest response rates, any analysis to assess the differential effectiveness of the PSD and GSD interventions that ignored mechanisms of missing data could potentially lead to biased estimates of population parameters. Therefore, the primary outcome evaluation for this trial was based on a ‘per-protocol’ analysis (i.e., participants who provided data on at least one occasion post-baseline). These analyses are, however, supplemented with ITT analyses for all outcome measures. The analysis of the G-SAS gambling symptom severity data involved maximum likelihood estimation (MLE), which assumes that missing data were missing at random (MAR). Sensitivity analyses were therefore conducted to assess for any departures in this MAR assumption using a pattern mixture approach with multiple imputation. Several plausible scenarios were explored using the user written Stata package mimix [64]. This type of sensitivity analysis enables the reader to critically appraise missing data assumptions relative to findings from a ‘per-protocol’ analysis.

A generalised mixed-effects model approach was used in the analysis of repeated measures for primary and secondary outcomes. Fixed effects in models were intervention group (GSD or PSD), time in categorical form, and interaction between intervention group and time. Random effects in the model were at study participant level and represented an upward or downward shift in the outcome measure from an overall regression line and rate of change over time. Linear and non-linear combinations of regression coefficients from mixed models were tested for treatment group effect at evaluation time points and estimated between-group mean differences are presented along with confidence intervals (CIs). Model fit was examined but there were no departures from the regression assumptions (see [49]). Linear mixed modelling was employed to compare treatment groups for continuous measures (G-SAS gambling symptom severity, G-SAS gambling urges, K6 psychological distress, EUROHIS quality of life). Because data for gambling frequency and gambling expenditure were non-normally distributed, the scores were collapsed into four ordered categories and analysed using mixed-effects ordered logistic regression. Similarly, because HSQ help-seeking data were non-normally distributed, the scores were dichotomised and analysed using mixed-effects binary logistic regression. The per-protocol analyses were also repeated after co-varying for low- and high-intensity help-seeking.

Effect sizes were presented as mean differences for continuous and normally distributed outcomes (G-SAS gambling symptom severity, G-SAS gambling urges, K6 psychological distress, EUROHIS-QOL quality of life) and odds ratios (ORs) for ordinal and categorical outcomes (gambling frequency, gambling expenditure, and help-seeking). Clinically significant change, as outlined by Jacobson and Truax [65], was also evaluated for G-SAS gambling symptom severity scores. At each evaluation, each participant’s status was defined as “recovered” if their evaluation score fell in the mild range or below (i.e., score of 20 or less), “improved” (final score corresponded to a reliable change, but fell into the dysfunctional range), “unchanged” (final score did not correspond to a reliable change), or “deteriorated” (final score corresponded to a reliable change in the negative direction).

Finally, a series of exploratory logistic regression models were performed to determine which factors predicted treatment outcome (i.e., unchanged/deteriorated cf. recovered/improved; the four categories of clinically significant change were dichotomised due to relatively small sample sizes), treatment engagement (completion of at least one program activity cf. no completion of at least one program activity), and post-baseline evaluation completion (non-completion of evaluation questionnaire cf. completion of evaluation questionnaire). Both univariate and multivariable models were calculated at 12 weeks, with 8 week data employed where 12 week data were not available, and at 24 months. Variable selection for regression models commenced with univariate analyses and then selected for model advancement based on *p* < 0.25 or *p* < 0.10 where sample size was modest [66]. To interpret effect sizes, ORs were calculated to represent the probability of experiencing one outcome category (e.g., “recovered/improved”) over the probability of experiencing the reference category (e.g., “unchanged/deteriorated”).

### 2.7. Sample Size

A total sample size of 100 participants at the final evaluation was needed to detect an effect size of 0.55 (Cohen’s d) for the primary outcome with statistical power of (1 − β) = 0.80 in a two-tailed test (*p* < 05). Taking into account a conservative dropout rate of 50%, the recruitment target was a sample of 200 participants (see [49]).

## 3. Results

### 3.1. Participants

**Figure 1:**
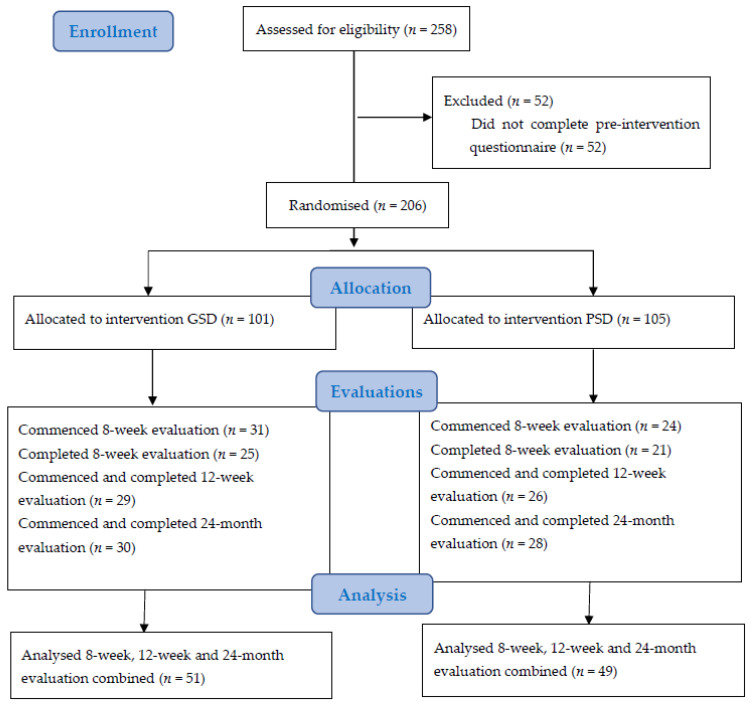
Consort flow diagram.

Participants were recruited into the trial from August 2015 until May 2016. Overall, 258 individuals provided informed consent online and commenced the baseline questionnaire. Of these, 206 individuals completed the baseline questionnaire and were randomly allocated to the GSD (*n* = 101) or PSD (*n* = 106) intervention. A total of 55 participants commenced the 8 week evaluation questionnaire, with 46 completing it (GSD: *n* = 25; PSD: *n* = 21). At 12 weeks, a total of 55 participants commenced and completed the questionnaire (GSD: *n* = 29; PSD: *n* = 26). At 24 months, a total of 58 participants commenced and completed the questionnaire (GSD: *n* = 30; PSD: *n* = 28). See Figure 1 below for Consolidated Standards of Reporting Trials (CONSORT) flow diagram. The majority of the 206 participants who completed the baseline questionnaire were recruited from Google advertising (63.1%), followed by Facebook advertising (12.6%), advertisements placed on the Gambling Help Online website (11.2%), other recruitment avenues not specified (6.3%), advertisements placed on other websites (3.9%), counsellor referral (1.9%), and advertisements placed on the Victorian Responsible Gambling Foundation website (1.0%).

### 3.2. Profile of GamblingLess Users

#### 3.2.1. Socio-Demographic Characteristics 

The socio-demographic profile of the sample is displayed in Table 1. Approximately two-thirds of participants were male (64.6%), were aged less than 40 years (63.6%), and used the internet for work, personal, education, or recreation reasons between 1 and 21 h a week (67.0%). The majority of the sample was born in Australia (76.2%) and most were employed full time (71.4%). Approximately one-third of the sample reported an annual personal net income of AUD$40,000 to $64,999 (33.5%), with an additional 39.8% earning higher than this income bracket. There were no significant differences in socio-demographic characteristics between participants allocated to the PSD and GSD interventions.

#### 3.2.2. Gambling Behaviour Characteristics

The gambling behaviour profile of the sample is displayed in Table S1 in the Supplementary Material. The most commonly endorsed problem gambling activities were EGMs (74.3%), followed by horse and greyhound racing (45.2%) and sports and events betting (27.2%). The mean PGSI problem gambling severity score was 17.8 (SD = 5.4), with almost all (96.1%) classified in the problem gambling category. The mean G-SAS gambling symptom severity score was 29.7 (SD = 7.7), with the majority in the moderate or severe categories (83.9%). The average total past-month gambling frequency was 13.3 days and the average total past-month gambling expenditure was AUD$1640, with the highest frequencies and expenditures reported for EGMs (6.0 days, AUD$1769), horse or greyhound racing (3.9 days, AUD$718) and sports and events betting (1.8 days, AUD$359). There were no significant differences in gambling behaviour characteristics between participants allocated to the PSD and GSD interventions.

#### 3.2.3. Psychological Characteristics 

The psychological profile of the sample is displayed in Table S2 in the Supplementary Material. The average K6 psychological distress score was 17.2 (SD = 5.6), with most participants classified in the moderate risk range or higher (71.8%). Half of the sample (50.0%) rated their quality of life as less than good. The majority of participants (80.6%) also screened positive for hazardous alcohol use on the AUDIT-3 and 18% screened positive for illegal drug use or a prescription medication for non-medical reasons. Participants reported high readiness and willingness to change their gambling, but lower levels of confidence in their ability to change. BSCQ gambling self-efficacy scores indicated that participants felt least confident to resist the urge to gamble in situations involving urges and temptations, unpleasant emotions, financial pressures, testing control over gambling, and social pressures to gamble. There were no significant differences in psychological characteristics between participants allocated to the PSD and GSD interventions.

#### 3.2.4. Treatment Characteristics 

The treatment characteristics of the sample are displayed in Table S3 in the Supplementary Material. Just under half of participants (48.5%) indicated that their goal was to quit gambling altogether, with an additional quarter of the sample indicating that their goal was to cut back (27.2%) or to quit (24.3%) problematic activities. Overall, 15.5% of participants engaged in high-intensity interventions in the previous month, the most common of which were financial counselling, talking to a professional (e.g., psychologist, psychologist, GP), and face-to-face gambling counselling. A slightly smaller proportion of participants engaged in low-intensity interventions in the previous month (10.7%), the most common of which involved counselling via the gambling helpline and online services. Moreover, nearly half (47.6%) engaged in at least one self-directed action in the previous month, the most common of which were talking to family members or friends, trying a self-help strategy like budgeting, and reading information on the Gambling Help Online website. There were no significant differences in treatment characteristics between participants allocated to the PSD and GSD interventions.

### 3.3. Effectiveness of the GamblingLess Program

There were no significant differences in baseline socio-demographic characteristics between PSD and GSD participants who completed the 8 week, 12 week, or 24 month evaluation (Table S4 in the Supplementary Material).

The patterns of missing data for both PSD and GSD participants were explored (G-SAS gambling symptom severity scores are provided in Table S5 in Supplementary Material as an illustration). Nearly half of the participants (48.5%) completed the 8 week, 12 week, or 24 month evaluation. The pattern of missing data for all secondary outcomes was similar to that provided for G-SAS gambling symptom severity. For the PSD intervention, only 6% of the entire sample completed all 4 assessments, with a further 16% completing baseline and at least two other assessments; and a further 24% completing baseline and at least one other assessment. For the GSD intervention, 14% of the entire sample completed all four assessments, with a further 11% completing baseline and at least two other assessments; and a further 26% completing baseline and at least one other assessment. There was no significant difference between the PSD (46.7%) and GSD (50.5%) conditions in completing the 8 week, 12 week, or 24 month evaluation (ꭓ^2^ = 0.30, *p* = 0.58).

Overall, 33.0% of participants completed an activity within the program. There was no significant difference between the PSD (31.4%) and GSD (34.7%) conditions in completing an activity within the program (ꭓ^2^ = 0.24, *p* = 0.62).

All patterns of results presented in this section were replicated when using an ITT analysis (Tables S6 and S7 in the Supplementary Material) and after controlling for help-seeking (Tables S8 and S9 in the Supplementary Material). 

#### 3.3.1. Primary Outcome: G-SAS Gambling Symptom Severity

The improvement in mean G-SAS scores in both the PSD and GSD intervention groups showed a similar trend: an initial fast improvement from baseline (moderate symptom severity) to 8 weeks (mild symptom severity), then a levelling-off effect at 12 weeks and 24 months (mild symptom severity) (Table 2). There was no significant intervention group × time interaction in G-SAS gambling symptom severity scores across the 24 month evaluation period (β = −0.16, 95% CI: −0.34, 0.03, *p* = 0.098) (Table 3). There was, however, a significant improvement in G−SAS gambling symptom severity scores within both the PSD (β = −4.42, 95% CI: −5.70, −3.15, *p* < 0.001) and GSD (β = −4.66, 95% CI: −5.90, −3.41, *p* < 0.001) conditions across the 24 month evaluation period (after controlling for age and sex), as shown in Table 4. As previously indicated, this pattern of results was also evident when using an ITT analysis, with the sensitivity analysis suggesting that trial findings at the 8 week, 12 week, and 24 month evaluations were mostly consistent with the MAR assumption from a substantive perspective (i.e., the nature of the trial design and interventions) (Table S10 in the Supplementary Material).

For clinically significant change calculated from G-SAS gambling symptom severity data from baseline to the 8 week evaluation (Table 5), 61.8% of participants were recovered or improved, with no statistically significant difference between the PSD (66.7%) and GSD (58.1%) conditions (ꭓ^2^ = 2.62, *p* = 0.45). From baseline to the 12 week evaluation, 67.2% of participants were recovered or improved, with no statistically significant difference between the PSD (65.4%) and GSD (69.0%) conditions (ꭓ^2^ = 7.52, *p* = 0.057). Finally, from baseline to the 24 month evaluation, 69.0% of participants were recovered or improved, with no statistically significant difference between the PSD (60.7%) and GSD (76.7%) conditions (ꭓ^2^ = 4.57, *p* = 0.10).

#### 3.3.2. Secondary Outcomes

Gambling urges. There was an initial fast reduction in mean G-SAS gambling urge subscale scores from baseline to 8 weeks for both groups, followed by a levelling-off effect for the PSD condition but further reductions in mean scores for the GSD condition at 12 and 24 months (Table 2). There was a significant intervention group × time interaction in G-SAS gambling urge scores, with GSD participants reporting greater improvement than PSD participants across the 24 month evaluation period (β = −0.09, 95% CI: −0.16, −0.02, *p* = 0.010) (Table 3). There was, however, a significant improvement in G-SAS gambling urge scores within both the PSD (β = −1.35, 95% CI: −1.81, −0.88, *p* < 0.001) and GSD (β = −1.33, 95% CI: −1.77, −0.89, *p* < 0.001) conditions across the 24 month evaluation period (Table 4).

Gambling Frequency. The average trajectory in gambling frequency across time was influenced by outliers, which were retained in the analyses. There was an initial fast reduction in mean scores from baseline to 8 weeks, a levelling-off effect at 12 weeks, then an increase at 24 months (Table 2). There was a significant intervention group × time interaction in gambling frequency, with GSD participants reporting greater improvement than PSD participants across the 24 month evaluation period (OR = 0.95, 95% CI: 0.91, 1.00, *p* = 0.046) (Table 3). There was, however, a significant improvement in gambling frequency within both the PSD (OR = 0.64, 95% CI: 0.46, 0.89, *p* = 0.007) and GSD (OR = 0.39, 95% CI: 0.27, 0.56, *p* < 0.001) conditions across the 24 month evaluation period (Table 4).

Gambling Expenditure. The average trajectories in gambling expenditure across time were similar to gambling frequency, with an initial improvement from baseline to the 8 week and 12 week evaluations, followed by a slight increase at the 24 month evaluation (Table 2). There was no significant intervention group × time interaction in gambling expenditure (OR = 0.99, 95% CI: 0.94, 1.04, *p* = 0.667) (Table 3), but there was a significant improvement in gambling expenditure within both the PSD (OR = 0.35, 95% CI: 0.23, 0.55, *p* < 0.001) and GSD (OR = 0.37, 95% CI: 0.25, 0.54, *p* < 0.001) conditions across the 24 month evaluation period (Table 4).

Psychological Distress. There was an initial fast reduction in mean K6 psychological distress scores from baseline (moderate risk) to 8 weeks (low risk) and then a levelling-off effect at 12 weeks (low risk) and 24 months (low risk) (Table 2). There was no significant intervention group × time interaction in K6 psychological distress (β = −0.03, 95% CI: −0.12, −0.07, *p* = 0.585) (Table 3), but there was a significant improvement in K6 psychological distress scores within both the PSD (β = −1.19, 95% CI: −1.76, −0.62, *p* < 0.001) and GSD (β = −1.78, 95% CI: −2.47, −1.09, *p* < 0.001) conditions across the 24 month evaluation period (Table 4).

Quality of Life. There was an initial improvement in mean EUROHIS quality of life item scores from baseline to the 8 week evaluation for both groups, after which there was a further small improvement for the GSD intervention and a small deterioration for the PSD intervention at the 12 week and 24 month evaluations (Table 2). There was no significant intervention group × time interaction in EUROHIS quality of life scores (β = 0.01, 95% CI: −0.01, 0.03, *p* = 0.210) (Table 3). There was, however, a significant improvement in EUROHIS quality of life scores within the GSD condition (β = 0.14, 95% CI: 0.03, 0.25, *p* = 0.013), but not the PSD condition (β = 0.05, 95% CI: −0.07, 0.17, *p* = 0.423) across the 24 month evaluation period (Table 4).

High-Intensity Help-Seeking. There was no improvement in high-intensity help-seeking for either group at the 8 week evaluation, but a small improvement in high-intensity help-seeking behaviour from 8 weeks to 12 weeks for both groups, which was followed by a levelling out for the PSD condition, but a reduction for the GSD condition at the 24 month evaluation (Table 2). Despite these differences, there was no significant intervention group × time interaction in high-intensity help-seeking (OR = 0.98, 95% CI: 0.91, 1.06, *p* = 0.660) (Table 3). There was also no significant improvement in high-intensity help-seeking within either the PSD (OR = 1.39, 95% CI: 0.84, 2.31, *p* = 0.199) or GSD (OR = 1.31, 95% CI: 0.84, 2.05, *p* = 0.239) conditions across the 24 month evaluation period (Table 4). 

Low-Intensity Help-Seeking. There was an initial large improvement in low-intensity help-seeking from baseline to the 8 week evaluation for the GSD intervention, which levelled off at the 12 week evaluation and deteriorated at the 24 month evaluation (Table 2). In contrast, there was a deterioration in the PSD intervention from baseline to the 8 week evaluation with a large increase at the 12 week evaluation and a small deterioration at the 24 month evaluation. Despite these differences, there was no significant intervention × time interaction in high-intensity help-seeking (OR = 0.93, 95% CI: 0.83, 1.03, *p* = 0.157) (Table 3). There was also no significant improvement in high-intensity help-seeking within either the PSD (OR = 1.23, 95% CI: 0.74, 2.06, *p* = 0.422) or GSD (OR = 1.33, 95% CI: 0.87, 2.04, *p* = 0.189) conditions across the 24 month evaluation period (Table 4).

### 3.4. Subgroups Benefiting Most from the GamblingLess Program

Exploratory binary logistic regressions were employed to investigate the prediction of clinically significant change in G-SAS gambling symptom severity at the 8 or 12 week evaluation (Table S11 in the Supplementary Material), clinically significant change in G-SAS gambling symptom severity at the 24 month evaluation (Table S12 in the Supplementary Material), treatment engagement as indicated by module activity completion (Table S13 in the Supplementary Material), and post-baseline evaluation completion (Table S14 in the Supplementary Material). G-SAS gambling symptom severity and G-SAS urges were not employed as predictors in clinically significant change analyses; and treatment engagement was not employed as a predictor in the module activity completion analysis.

#### 3.4.1. Predictors of Short-Term Treatment Outcomes 

Overall, 63.8% of participants recovered or improved based on clinically significant change from baseline to the 8 or 12 week evaluation on G-SAS gambling symptom severity. The referent category was participants who were unchanged or deteriorated according to their G-SAS gambling symptom severity clinically significant change indices. In the univariate models, gambling problems on EGMs only (OR = 5.50, (*p* = 0.007) and scores on importance rulers (OR = 1.81, *p* = 0.047) were statistically significant. These predictors remained significant in the multivariable model: gambling problems on EGMs only (OR = 12.83, *p* = 0.014) and baseline importance ruler scores (OR = 1.79, *p* = 0.046).

#### 3.4.2. Predictors of Long-Term Treatment Outcomes 

Overall, 69.0% of participants recovered or improved based on clinically significant change from baseline to the 24 month evaluation on G-SAS gambling symptom severity. The referent category was participants who were unchanged or deteriorated according to their G-SAS gambling symptom severity clinically significant change indices. The only significant univariate predictor of long-term outcomes was average weekly internet use (OR = 1.45, *p* = 0.025). In the multivariable model, this variable remained significant (OR = 1.59, *p* = 0.015). However, sex was also a significant predictor of long-term treatment outcomes in the multivariate model, whereby the odds of recovery or improvement decreased by a factor of 0.19 (*p* = 0.030) for male participants.

#### 3.4.3. Predictors of Treatment Engagement

Overall, 33% of participants completed an activity within the program. The classification of treatment engagement was established on a participant completing at least one module activity, while the referent category was participants who had not completed any module activity. In the univariate models, age group (OR = 1.24, *p* = 0.001), average weekly internet use (OR = 1.20, *p* = 0.013), and past-month self-directed actions (OR = 2.68, *p* = 0.001) were statistically significant. In the multivariable model, all of these variables remained statistically significant: age group (OR = 1.28, *p* = 0.001), average weekly internet use (OR = 1.29, *p* = 0.002), and past-month self-directed actions (OR = 2.93, *p* = 0.001). Moreover, BSCQ self-efficacy was also a significant predictor of module completion in the multivariate model (OR = 1.02, *p* = 0.031). 

#### 3.4.4. Predictors of Post-Baseline Evaluation Completion

Nearly half of the participants (48.5%) completed the 8 week, 12 week, or 24 month evaluation. Classification as an “evaluation completer” was based on a participant completing at least one set of evaluations (i.e., 8 week, 12 week, or 24 month evaluation), while the referent category was participants who had completed the pre-intervention evaluation only. In the univariate models, G-SAS gambling urges (OR = 0.90, *p* = 0.034), past-month high-intensity help-seeking (OR = 2.65, *p* = 0.018) and treatment engagement (OR = 1.07, *p* = 0.003) were statistically significant predictors of post-baseline evaluation completion. In the multivariable model, these predictors remained significant: G-SAS gambling urges (OR = 0.90, *p* = 0.047), past-month high-intensity help-seeking (OR = 2.93, *p* = 0.015), and treatment engagement (OR = 1.06, *p* = 0.010). 

## 4. Discussion

This study compares the effectiveness of GamblingLess, an internet-based cognitive-behavioural therapeutic program designed to assist people with gambling problems, with and without guidance. This study is the first to explore the efficacy of adding guidance to this therapeutic modality for gambling problems. Secondary aims were to examine the profile of end-users and subgroups of gamblers who can most benefit from the program.

### 4.1. Effectiveness of the GamblingLess Program

The findings generally support the delivery of the GamblingLess program, with and without guidance. Participants in both treatment conditions reported significant improvements on almost all outcome measures (gambling symptom severity, gambling urges, gambling frequency, gambling expenditure, and psychological distress) across the 24 month evaluation period. This pattern of results was replicated when using ITT analyses and after controlling for other help-seeking. These findings are consistent with previous promising results resulting from other PSD internet-based therapeutic interventions for gambling [30,31,32,33,34]. Moreover, almost 70% of participants reported recovery or improvement on gambling symptom severity at the 24 month evaluation period. Specifically, 56.9% of participants were classified as recovered, 12.1% were classified as improved, and 31% were classified as unchanged. These rates of clinically significant change based on gambling symptom severity compare favourably to those identified for face-to-face MI/CBT gambling interventions [67,68]. For example, it was noted that 2.6% of participants completing a 4-session face-to-face MET plus CBT intervention were classified as recovered, 47.4% of participants were classified as improved, and 50% were classified as unchanged [68]. These findings indicate that a considerable proportion of GamblingLess users displayed clinically significant changes in the severity of their gambling symptoms. There was, however, no within-group improvement on either low- or high-intensity help-seeking for either treatment condition, indicating either that participants were satisfied with the level of help they were currently receiving or that help-seeking requires additional emphasis in the program. 

The primary aim of this study was to compare the effectiveness of the program delivered under PSD and GSD conditions. There were few statistically significant differences in the efficacy of the PSD and GSD interventions across the 24 month evaluation period. The two treatment conditions did not differ on gambling symptom severity, gambling urges, gambling expenditure, psychological distress, or additional help-seeking. There were also no differences in the PSD and GSD conditions in completing at least one module activity (treatment engagement) or completing at least of set of evaluations (i.e., 8 week, 12 week, or 24 month evaluation). While these findings seem to provide preliminary evidence that the addition of guidance does not seem to substantially improve outcomes for the GamblingLess program, other findings from the trial are more promising. The GSD intervention was more effective in reducing gambling urges and gambling frequency than the PSD intervention. Although there was no significant difference between the GSD and PSD conditions on quality of life, participants in the GSD condition, but not the PSD condition, reported improved quality of life across the evaluation period. Moreover, a considerably higher proportion of GSD participants (77%) than PSD participants (60.7%) reported being recovered or improved on gambling symptom severity at the 24 month evaluation, a difference that only just failed to reach statistical significance. These findings are certainly more promising than previously reported [42], which evaluated the addition of guidance to a CBT self-help workbook among non-help-seeking people with gambling problems naturalistically recruited in their gambling environments. They are also more consistent with the findings in other fields, whereby GSD interventions are more effective than PSD interventions [14,22], although the difference in effect is probably less pronounced than previously thought [22].

### 4.2. Profile of GamblingLess Users

A secondary aim of this study was to explore the profile of GamblingLess users. Participants in this study were mostly young male Australian-born gamblers who were employed full time and had relatively high annual personal net incomes. They most commonly reported having issues with EGMs, but issues with horse, harness or greyhound racing and sports and events betting were also commonly reported. Almost all participants were classified in the problem gambling category of the PGSI. This demographic and gambling profile is consistent with that reported in previous studies evaluating internet-based therapeutic interventions [29,30,31,32,33,34,35,36,37,38,39,40,41] and is very similar to gamblers using the national online gambling service [43,44]. These findings may indicate that young males are attracted to online modalities, in preferences to other service modalities, such as face-to-face counselling. They suggest that peers or professionals supporting gamblers in the use of these programs require an understanding of gambling activities involving strategy, as well as EGM gambling. They also suggest that internet-based therapeutic interventions attract gamblers who experience problems at the severe end of the gambling continuum, which is inconsistent with the view that the adoption of self-help models reflects a ‘stepped care’ approach, whereby “low-intensity” evidence-based interventions are provided to a proportion of lower- or at-risk groups in the first instance [16,69]. It is evident that the target audience for different internet-based interventions needs to be articulated more clearly in the future and that further effort is required to target gamblers with lower problem gambling severities [70].

Users of the GamblingLess program reported considerable psychological dysfunction, with high rates of psychological distress, poor quality of life, hazardous alcohol use, and illegal drug use or prescription medication for non-medical reasons, which is consistent with previous treatment-seeking literature [71,72,73,74]. These findings highlight the possible need for online self-directed programs for gambling to routinely screen and assess for psychiatric comorbidity and provide referral or resources that adequately address these comorbid issues [75,76,77,78]. While the provision of such resources has the potential to allow for a more flexible, cost-effective and individually tailored approach, several studies that have added supplementary content addressing these issues to internet-based therapeutic gambling interventions have not demonstrated significantly improved outcomes [32,33]. Users of the GamblingLess program also reported generally high readiness and willingness to change, but very low confidence in their ability to limit or stop gambling or to resist the urge to gamble in high-risk situations. This readiness to change profile is the same as that reported by gamblers using the national online gambling service [61]. These findings support the content of the GamblingLess program, in which the focus was less on motivational approaches to increase readiness to change and more on the development of self-efficacy in relation to managing gambling urges and coping with high-risk situations. 

Users of the GamblingLess program were required to select their goal of treatment, in recognition of increasing evidence that offering non-abstinence goals may provide a more realistic and appealing option to some gamblers, particularly for those who doubt their ability to abstain [79,80,81]. At baseline, more than half of the participants selected a reduced gambling goal, in preference to complete abstinence, which is consistent with research conducted in Australian face-to-face [79,82] and online [43] services. It is apparent that, at least in the Australian context, non-abstinence goals are relatively popular for gamblers accessing internet-based therapeutic interventions. Finally, although a small proportion of users accessed high- and low-intensity interventions, in addition to the GamblingLess program, nearly half engaged in self-directed actions in the previous month. This pattern of help-seeking behaviour, which reflects that of the users of the national online gambling service [43], suggest these gamblers concurrently attempt to access multiple help options, particularly self-help options. These findings support the attempt by the GamblingLess program, and other internet-based therapeutic interventions, in encouraging users to seek formal, as well as informal, sources of help and providing contact details for multiple help options.

### 4.3. Subgroups Benefiting Most from the GamblingLess Program

There were few significant predictors of short- or long-term clinically significant change on gambling symptom severity scores, treatment engagement, or completion of post-baseline evaluations. These findings are consistent with previous studies, which have identified few clear predictors of outcomes following gambling treatment [45,47] and internet-based therapeutic interventions [46]. Gamblers who gambled on EGMs only and for whom limiting or stopping gambling was high on their list of priorities were most likely to benefit from the intervention in the short term, but female users with high average weekly internet use were most likely to benefit in the long term. While these findings are consistent with systematic review evidence [45] that readiness to change is associated with gambling treatment outcomes, they contrast with other evidence from the same review suggesting that preferred gambling activity is not associated with outcomes, treatment goal has demonstrated mixed findings, and that male sex is one of the strongest predictors of outcomes.

Older gamblers with high self-efficacy, who engaged in self-directed actions and had good access to the internet, were most likely to complete a module activity. Again, these findings are somewhat inconsistent with systematic review evidence that has concluded that age has demonstrated mixed findings and that self-efficacy has too few studies from which to draw a firm conclusion [46]. Finally, gamblers with high gambling urges were less likely to complete a post-baseline evaluation, while participants who sought additional high-intensity interventions and had high treatment engagement were more likely complete an evaluation. While prior treatment experience and treatment motivation have previously not been associated with the outcomes of psychological gambling treatments [47], there are too few studies from which conclusions can be drawn.

While the predictors of treatment outcomes, treatment engagement, or completion of post-baseline evaluations identified in this study are clinically logical, it is clear that few are consistent with those identified in previous literature exploring the factors associated with the outcomes of internet-based therapeutic gambling interventions [29,34,35,38], internet-based therapeutic interventions [46] or psychological gambling treatments more generally [45,47]. This emerging field of research would therefore benefit from future research that examines the moderators and predictors of gambling outcomes from internet-based therapeutic gambling interventions so that their evaluation can be safeguarded against bias [83] and they can be personalised to meet individual needs [45].

### 4.4. Study Limitations

When considering the practical implications of the findings of this study, it is important to note several methodological limitations. Like most online self-guided psychological interventions [84,85,86,87], low engagement was a limitation of the intervention, with only one-third of users (33%) completing one activity in the program. Moreover, the addition of guidance did not seem to enhance the rate of treatment engagement. The low rate of activity completion in this study is of concern given that poor engagement is related to reduced treatment efficacy [84,88,89,90] as it limits exposure to the full program or the required “dosage” of treatment [85]. Future acceptability and feasibility evaluations are required to explore the reasons why some users do not complete any of the intervention activities, as this information could be used to refine the program [85]. Similarly, a limitation of this study included low data collection rates, with nearly half of the participants completing at least one post-baseline evaluation. This evaluation completion rate is consistent with previous studies of online psychological interventions which have been found to be substantially lower than in conventional trials and interventions [83]. Further, the addition of guidance did not seem to enhance the rate of post-baseline evaluation completion. Although attempts were made to increase data collection rates, including multiple and varying modes of contact and participant remuneration, missing data due to the evaluation attrition resulted in relatively smaller samples and therefore likely reduced the statistical power of this study for both the primary and secondary analyses. It is clear that achieving desirable follow-up rates remains a significant challenge to internet-based intervention trials [83].

Moreover, the overall effectiveness of the intervention can only be inferred from correlational findings based on changes in outcome measures over time rather than direct evaluation with a control group through the randomised component of the study design. The high overall rate of improvement on the G-SAS, in combination with the low rate of activity completion in this intervention, implies that some participants naturally recovered or improved due to other treatments they were receiving, reactivity to the research assessments and contact, or regression to the mean. A passive control group was not included in this study for several reasons. First, there is an increasing awareness of the ethical concerns in relation to providing untreated control groups, particularly when longer-term follow-up evaluations are planned. Second, demonstrating the superiority of a GSD intervention over an untreated control group did not seem warranted given that they are the gold standard of self-directed interventions, with established long-term effectiveness for many disorders [19] (Wagner et al., 2013). Nonetheless, randomised trials employing a no-intervention control group or some other naturalistic treatment-as-usual or standard treatment would be informative.

Finally, in this study, there was a reliance upon self-report measures with no clinical diagnostic procedure to confirm the results, which may have resulted in self-report biases. While corroboration of self-report has been identified as important in determining the accuracy of gambling behaviour and impacts [91], the anonymous nature of this mode of intervention makes this opportunity more difficult. Influences including location (e.g., work, home), physical factors (e.g., intoxication), and psychological condition (e.g., distress) may impact administration validity of self-report measures [92]. Moreover, a single item measure was employed to assess quality of life and a non-validated measure was employed to assess help-seeking behaviour, neither of which may have been sensitive to change.

### 4.5. Clinical Implications

Despite these limitations, this study highlights the potential effectiveness of internet-based therapeutic gambling interventions. Such interventions can be integrated into standard service delivery models to increase the accessibility of evidence-based treatment for people with gambling problems and can be offered within a suite of available interventions to ensure gamblers have access to the service most likely to suit their needs. The current evidence for MI and CBT in the treatment of problem gambling [1,2,3,4], in combination with the promising results from this and other studies, suggest that the delivery of iCBT is appropriate. Although initial efforts have not improved outcomes [33], users may benefit from supplementary content or additional resources related to mental health or substance use. Moreover, the low rate of engagement suggests that the intervention may benefit from additional strategies to enhance engagement, such as the inclusion of follow-up modules that make long-term support possible, face-to-face contact before referral into the program, frequent reminder emails, rewards and bonuses, tailored emails and website content, and enhancement of the graphics and interactivity [87,93].

It should be noted that this intervention was developed to emulate the intensity and depth of a face-to-face psychological intervention. There is evidence that there is a strong dose–response relationship between the number and intensity of counselling sessions and treatment outcomes [94,95]. Moreover, in the gambling field, high-intensity self-directed interventions, defined as structured therapy programs with six or more sessions or modules delivered over the internet and over a period of several weeks, including homework assignments, participation in online discussion groups, or interactive exercises, are associated with much better post-treatment outcomes than lower-intensity self-directed interventions [8]. There is, however, also evidence that treatment engagement and attrition are associated with the intensity and extensity of internet-based intervention content [96,97,98]. Although the intervention would likely benefit from a reduction in the number of activities, content brevity must be balanced with therapeutic dosing so that the burden of participation does not lead to disengagement but the activities contain sufficient detail to be therapeutic and address the presenting issue [87]. Accordingly, this trial with an adjunctive in-depth methodology has informed the development of several more brief and targeted online and mobile interventions [99,100,101,102,103,104].

This study also provides promising results in relation to the addition of guidance to internet-based therapeutic gambling interventions, with further research appearing warranted. Findings from other fields can inform future research in this area. In particular, it would be of interest to evaluate the influence of the modality of guidance (e.g., telephone, email, SMS, chat, face to face), the amount of guidance required, over what duration guidance should be provided, and the degree of personalisation required [20]. Future research is required to investigate the efficacy of the program delivered in combination with peer support or coaching [105,106] and determine the degree to which the addition of therapeutic content delivered by trained professionals would improve treatment outcomes [107,108]. A greater understanding of when and who benefits when guidance or support is added is needed given that unguided interventions are effective and can be delivered at lower cost [22,28,109].

## 5. Conclusions

This study is the first to evaluate the efficacy of adding guidance to an internet-based therapeutic gambling intervention. The findings provide support for the delivery of this intervention and suggest that there is some benefit to adding guidance to this intervention. Participants in both treatment conditions reported significant improvements on almost all outcome measures across the 24 month evaluation period, even after using ITT analyses and controlling for other help-seeking, as well as clinically significant changes in gambling symptom severity. Compared to the PSD intervention, the GSD intervention resulted in additional improvements to gambling urges and frequency, within-group change in quality of life, and somewhat higher rates of clinically significant change in gambling symptom severity. Further research investigating the addition of guidance to internet-based therapeutic gambling interventions therefore appears warranted. Given the efficacy and lower costs associated with the delivery of unguided interventions, additional research is required to establish when and for whom human support adds value.

## Figures and Tables

**Table 1:** Baseline sample socio-demographic characteristics.

Socio-Demographic Characteristic	Pure Self-Directed (*n* = 105)	Guided Self-Directed (*n* = 101)	Total (*n* = 206)
Sex (*n*, % male)	67 (63.8)	66 (65.3)	133 (64.6)
Age group in years (*n*, %)			
18–24	17 (16.2)	24 (23.8)	41 (19.9)
25–29	17 (16.2)	20 (19.8)	37 (18.0)
30–34	15 (14.3)	9 (8.9)	24 (11.7)
35–39	18 (17.1)	11 (10.9)	29 (14.1)
40–44	11 (10.5)	9 (8.9)	20 (9.7)
45–49	6 (5.7)	10 (9.9)	16 (7.8)
50–54	10 (9.5)	10 (9.9)	20 (9.7)
55+	11 (10.5)	8 (7.9)	19 (9.2)
Country of birth (*n*, % Australia)	81 (77.1)	76 (75.3)	157 (76.2)
Employment (*n*, %)			
Work full time	77 (73.3)	70 (69.3)	147 (71.4)
Work part time/casual	13 (12.4)	22 (21.8)	35 (17.0)
Unemployed	5 (4.8)	2 (2.0)	7 (3.4)
Full time student	3 (2.9)	1 (1.0)	4 (1.9)
Full-time home duties	1 (1.0)	1 (1.0)	2 (1.0)
Retired	3 (2.9)	0 (0)	3 (1.5)
Sick or disability pension	2 (1.9)	4 (4.0)	6 (2.9)
Other	1 (1.0)	1 (1.0)	2 (1.0)
Annual personal net income (*n*, %)			
<AUD$25,000	13 (12.4)	10 (9.9)	23 (11.2)
AUD$25,000–$39,999	18 (17.1)	14 (13.9)	32 (15.5)
AUD$40,000–$64,999	28 (26.7)	41 (40.6)	69 (33.5)
AUD$65,000–$79,999	20 (19.1)	17 (16.8)	37 (18.0)
AUD$80,000–$129,999	18 (17.1)	15 (14.9)	33 (16.0)
AUD$130,000+	8 (7.6)	4 (4.0)	12 (5.8)
Internet use (hours) (*n*, %) ^a^			
Less than 1	6 (5.7)	2 (2.0)	8 (3.9)
1–7	25 (23.8)	19 (18.8)	44 (21.4)
8–14	29 (27.6)	28 (27.7)	57 (27.7)
15–21	15 (14.3)	22 (21.8)	37 (18.0)
22–28	7 (6.7)	7 (6.9)	14 (6.8)
29–35	8 (7.6)	6 (5.9)	14 (6.8)
36–42	3 (2.9)	9 (8.9)	12 (5.8)
More than 42	12 (11.4)	8 (7.9)	20 (9.7)

^a^ Based on average weekly use for work/personal/education/recreation.

**Table 2:** Descriptive statistics across time points for all primary and secondary outcomes.

Outcome	Baseline		8 Weeks		12 Weeks		24 Months	
	PSD (*n* = 50)	GSD (*n* = 51)	PSD (*n* = 22)	GSD (*n* = 28)	PSD (*n* = 26)	GSD (*n* = 29)	PSD (*n* = 28)	GSD (*n* = 30)
G-SAS gambling symptom severity ^a^	30.12 (7.92)	27.94 (7.97)	17.50 (11.91)	19.45 (11.82)	20.42 (10.63)	15.14 (9.91)	19.79 (10.90)	13.87 (10.88)
G-SAS gambling urges ^a^	10.18 (2.84)	9.06 (2.77)	6.13 (4.71)	6.94 (4.17)	7.15 (4.10)	5.14 (3.54)	6.89 (3.95)	3.93 (3.94)
Gambling frequency ^b^	13.20 (11.57)	14.43 (19.64)	6.14 (7.89)	6.82 (8.58)	7.88 (8.50)	3.72 (4.71)	14.89 (17.58)	11.57 (21.23)
Gambling expenditure (AUD$) ^b^	3590 (6923)	2579 (5096)	511 (1123)	664 (1037)	747 (973)	272 (352)	1511 (2497)	1027 (1657)
K6 psychological distress ^a^	16.74 (5.92)	17.24 (5.56)	12.50 (4.98	13.93 (5.96)	14.00 (6.01)	12.66 (6.30)	13.64 (4.98)	12.17 (4.57)
EUROHIS quality of life ^a^	3.32 (1.15)	3.35 (1.04)	3.68 (0.84)	3.57 (1.00)	3.31 (1.01)	3.79 (0.90)	3.43 (1.03)	3.83 (0.79)
HSQ high-intensity help-seeking ^a,b^	0.32 (0.68)	0.31 (0.73)	0.32 (0.57)	0.32 (0.72)	0.58 (0.95)	0.52 (0.78)	0.46 (0.88)	0.20 (0.41)
HSQ low-intensity help-seeking ^a,b^	0.14 (0.40)	0.20 (0.57)	0.05 (0.21)	0.32 (0.48)	0.27 (0.53)	0.28 (0.59)	0.18 (0.55)	0.10 (0.55)

^a^ G-SAS: Gambling Symptom Assessment Scale; K6: Kessler 6 Psychological Distress Scale; EUROHIS (first item); HSQ: Help-Seeking Questionnaire. ^b^ Past 30 days.

**Table 3:** Per-protocol between-group comparison of PSD and GSD conditions on primary and secondary outcomes ^a^.

Outcome	Unadjusted Estimate (SE)				Estimated Between-Group Difference (95% CI)	*p*
	Baseline	8 Weeks	12 Weeks	24 Months		
G-SAS gambling symptom severity ^b^	-	−0.32 (0.19)	−0.47 (0.29)	−3.78 (2.29)	−0.16 (−0.34, 0.03) ^c^	0.098
G-SAS gambling urges ^b^	-	−0.18 (0.07)	−0.27 (0.10)	−2.17 (0.84)	−0.09 (−0.16, −0.02) ^c^	0.010
Gambling frequency	-	−	−	−	0.95 (0.91, 1.00) ^d^	0.046
Gambling expenditure	-	−	−	−	0.99 (0.94, 1.04) ^d^	0.667
K6 psychological distress ^b^	-	−0.05 (0.10)	−0.08 (0.15)	−0.64 (1.18)	−0.03 (−0.12, 0.07) ^c^	0.585
EUROHIS quality of life ^b^	-	0.02 (0.02)	0.03 (0.03)	0.26 (0.21)	0.01 (−0.01, 0.03) ^c^	0.210
HSQ high-intensity help-seeking ^b^	-	−	−	−	0.98 (0.91, 1.06) ^e^	0.660
HSQ low-intensity help-seeking ^b^	-	−	−	−	0.93 (0.83, 1.03) ^e^	0.157

^a^ Adjusted for participant age and gender. ^b^ G-SAS: Gambling Symptom Assessment Scale; K6: Kessler 6 Psychological Distress Scale; EUROHIS (first item); HSQ: Help-Seeking Questionnaire. ^c^ Linear mixed regression models. ^d^ Mixed-effects ordered logistic regression models—estimates reported as ORs (reference category = PSD). ^e^ Mixed-effects logistic regression models—estimates reported as ORs (reference category = PSD).

**Table 4:** Per-protocol within-group change for PSD and GSD conditions in primary and secondary outcomes ^a^.

Outcome	Treatment Group	Unadjusted Estimate (SE)				Estimated Within-Group Difference (95% CI)	*p*
		Baseline	8 Weeks	12 Weeks	24 Months		
G-SAS gambling symptom severity ^b^	PSD	29.69 (1.39)	21.50 (1.32)	17.90 (1.59)	18.43 (1.85)	−4.42 (−5.70, −3.15) ^c^	<0.001
	GSD	27.92 (1.35)	19.29 (1.22)	15.48 (1.48)	13.84 (1.68)	−4.66 (−5.90, −3.41) ^c^	<0.001
G-SAS gambling urges ^b^	PSD	10.05 (0.52)	7.55 (0.50)	6.45 (0.60)	6.54 (0.65)	−1.35 (−1.81, −0.88) ^c^	<0.001
	GSD	9.10 (0.47)	6.63 (0.42)	5.53 (0.52)	3.95 (0.64)	−1.33 (−1.77, −0.89) ^c^	<0.001
Gambling frequency	PSD	-	−0.82 (0.31)	−1.17 (0.44)	−0.89 (0.46)	0.64 (0.46, 0.89) ^d^	0.007
	GSD	-	−1.76 (0.35)	−2.52 (0.50)	−1.38 (0.48)	0.39 (0.27, 0.56) ^d^	<0.001
Gambling expenditure	PSD	-	−1.91 (0.41)	−2.75 (0.58)	−1.77 (0.60)	0.35 (0.23, 0.55) ^d^	<0.001
	GSD	-	−1.84 (0.35)	−2.64 (0.50)	−1.70 (0.54)	0.37 (0.25, 0.54) ^d^	<0.001
K6 psychological distress ^b^	PSD	16.57 (0.77)	14.36 (0.75)	13.38 (0.85)	12.39 (0.91)	−1.19 (−1.76, −0.62) ^c^	<0.001
	GSD	17.19 (0.76)	13.89 (0.70)	12.43 (0.85)	12.11 (0.94)	−1.78 (−2.47, −1.09) ^c^	<0.001
EUROHIS quality of life ^b^	PSD	3.35 (0.15)	3.44 (0.14)	3.48 (0.16)	3.57 (0.18)	0.05 (−0.07, 0.17) ^c^	0.423
	GSD	3.35 (0.13)	3.61 (0.12)	3.73 (0.14)	3.85 (0.16)	0.14 (0.03, 0.25) ^c^	0.013
HSQ high-intensity help-seeking ^b^	PSD	−2.12 (0.64)	−1.51 (0.57)	−1.24 (0.65)	−1.88 (0.75)	1.39 (0.84, 2.31) ^e^	0.199
	GSD	−1.87 (0.52)	−1.38 (0.43)	−1.17 (0.51)	−1.96 (0.65)	1.31 (0.84, 2.05) ^e^	0.239
HSQ low-intensity help-seeking ^b^	PSD	−2.26 (0.55)	−1.88 (0.45)	−1.71 (0.55)	−2.27 (0.72)	1.23 (0.74, 2.06) ^e^	0.422
	GSD	−1.84 (0.47)	−1.33 (0.37)	−1.12 (0.44)	−3.57 (1.11)	1.33 (0.87, 2.04) ^e^	0.189

^a^ Adjusted for participant age and gender. ^b^ G-SAS: Gambling Symptom Assessment Scale; K6: Kessler 6 Psychological Distress Scale; EUROHIS (first item); HSQ: Help-Seeking Questionnaire. ^c^ Linear mixed regression models. ^d^ Mixed-effects ordered logistic regression models-estimates reported as ORs. ^e^ Mixed-effects logistic regression models-estimates reported as ORs.

**Table 5:** Clinically significant change from baseline based on G-SAS gambling symptom severity.

**8 weeks**	**PSD (*n* = 24)**	**GSD (*n* = 31)**	**Total (*n* = 55)**
Recovered	14 (58.3)	12 (38.7)	26 (47.3)
Improved	2 (8.3)	6 (19.4)	8 (14.6)
Unchanged	7 (29.2)	12 (38.7)	19 (34.6)
Deteriorated	1 (4.2)	1 (3.2)	2 (3.6)
**12 weeks**	**PSD (*n* = 26)**	**GSD (*n* = 29)**	**Total (*n* = 55)**
Recovered	9 (34.6)	18 (62.1)	27 (49.1)
Improved	8 (30.8)	2 (6.9)	10 (18.2)
Unchanged	9 (34.6)	8 (27.6)	17 (30.9)
Deteriorated	0 (0.0)	1 (3.5)	1 (1.8)
**24 months**	**PSD (*n* = 28)**	**GSD (*n* = 30)**	**Total (*n* = 58)**
Recovered	12 (42.9)	21 (70.0)	33 (56.9)
Improved	5 (17.9)	2 (6.7)	7 (12.1)
Unchanged	11 (39.3)	7 (23.3)	18 (31.0)
Deteriorated	0 (0.0)	0 (0.0)	0 (0.0)

## Data Availability

The data presented in this study are available on request from the corresponding author. The data are not publicly available due to privacy and ethical restrictions.

## Supplementary Materials

The following are available online at https://www.mdpi.com/article/10.3390/jcm10112224/s1, Table S1: Baseline sample gambling behaviour characteristics; Table S2: Baseline sample psychological characteristics; Table S3: Baseline sample treatment characteristics; Table S4: Baseline differences between PSD and GSD participants who completed the 8 week, 12 week or 24 month evaluation; Table S5: Patterns of missing data for G-SAS gambling symptom severity; Table S6: Intention-to-treat between-group comparison of PSD and GSD conditions on primary and secondary outcomes; Table S7: Intention-to-treat within-group change for PSD and GSD conditions in primary and secondary outcomes; Table S8: Per-protocol between-group comparison of PSD and GSD conditions on primary and secondary outcomes after controlling for help-seeking; Table S9: Per-protocol within-group change for PSD and GSD conditions in primary and secondary outcomes after controlling for help-seeking; Table S10: Estimated effect of GSD intervention (vs PSD intervention) from per-protocol, multiple imputation under MAR, multiple imputation under LMCF, multiple imputation under J2R, and multiple imputation under CIR; Table S11: Exploratory univariate and multivariate logistic regression models of factors associated with clinically significant change on G-SAS gambling symptom severity at 8 or 12 week evaluation; Table S12: Exploratory univariate and multivariate logistic regression models of factors associated with clinically significant change on G-SAS gambling symptom severity at 24 month evaluation; Table S13: Exploratory univariate and multivariate logistic regression models of factors associated with module activity completion; Table S14: Exploratory univariate and multivariate logistic regression models of factors associated with post-baseline evaluation completion.
