# Supplementary material for: GamblingLess: A Randomised Trial Comparing Guided and Unguided Internet-Based Gambling Interventions
Source: J Clin Med. 2021 May 21;10(11):2224. doi: 10.3390/jcm10112224 (PMC8196610; doi:10.3390/jcm10112224)
Supplement: Supplementary file 1 [file jcm-10-02224-s001.zip › jcm-1177368-supplementary.pdf]

Supplementary Material

Table S1. Baseline sample gambling behaviour characteristics

**Gambling behaviour characteristic**	**Pure self-directed (n = 105)**	**Guided self-directed (n = 101)**	**Total (n = 206)**
Problematic gambling activities (n, %) [a]			
EGMs	77 (73.3)	76 (75.3)	153 (74.3)
Casino table games (e.g., blackjack)	15 (14.3)	18 (17.8)	33 (16.0)
Horse, harness or greyhound racing	54 (51.4)	39 (38.6)	93 (45.2)
Sports and events betting	33 (31.4)	23 (22.8)	56 (27.2)
Number games (e.g., lotteries, keno, bingo)	12 (11.4)	13 (12.9)	25 (12.1)
Informal private games	5 (4.8)	4 (4.0)	9 (4.4)
PGSI problem gambling severity category (n, %) [b]			
Problem gambling	102 (97.1)	96 (95.0)	198 (96.1)
Moderate risk gambling	2 (1.9)	5 (5.0)	7 (3.4)
Low risk gambling	0 (0.0)	0 (0.0)	0 (0.0)
Non-problem gambling	1 (1.0)	0 (0.0)	1 (0.5)
G-SAS gambling symptom severity category (n, %) [b]			
Extreme	7 (6.7)	7 (6.9)	14 (6.8)
Severe	44 (41.9)	28 (27.7)	72 (35.0)
Moderate	45 (42.9)	56 (55.5)	101 (49.0)
Mild	7 (6.7)	9 (8.9)	16 (7.8)
Gambling frequency (days) (M, SD) [c]			
EGMs	5.3 (6.5)	6.6 (7.3)	6.0 (6.9)
Casino table games (e.g., blackjack)	0.1 (0.7)	0.4 (2.6)	0.3 (1.9)
Horse, harness or greyhound racing	4.4 (7.6)	3.5 (7.1)	3.9 (7.3)
Sports and events betting	1.3 (4.1)	2.3 (6.5)	1.8 (5.4)
Number games (e.g., lotteries, keno, bingo)	1.3 (3.9)	2.0 (5.7)	1.6 (4.9)
Informal private games	0.0 (0.1)	0.3 (2.5)	0.2 (1.8)
Total gambling frequency	11.9 (11.5)	14.7 (16.9)	13.3 (14.5)
Gambling expenditure (AUD$) (M, SD) [c]			
EGMs	1742 (4340)	1798 (3440)	1769 (3916)
Casino table games (e.g., blackjack)	46 (249)	30 (151)	38 (207)
Horse, harness or greyhound racing	967 (3240)	459 (1026)	718 (2430)
Sports and events betting	328 (2458)	391 (2994)	359 (2728)
Number games (e.g., lotteries, keno, bingo)	56 (230)	51 (230)	54 (229)
Informal private games	0 (0)	4 (40)	2 (28)
Total gambling expenditure	1953(4359)	1315 (3459)	1640 (3947)

[a] Participants could endorse more than one option

[b] PGSI: Problem Gambling Severity Index; G-SAS: Gambling Symptom Assessment Scale

[c] Based on previous 30 days

Table S2. Baseline sample psychological characteristics

Psychological characteristic	Pure self-directed (n = 105)	Guided self-directed (n = 101)	Total (n = 206)
K6 psychological distress category (n, %) [a]			
Very high risk	7 (6.7)	12 (11.9)	19 (9.2)
High risk	33 (31.4)	24 (23.8)	57 (27.7)
Moderate risk	37 (35.2)	35 (34.6)	72 (34.9)
Low risk	28 (26.7)	30 (29.7)	58 (28.2)
EUROHIS quality of life (n, %) [a, b]			
Very poor	6 (5.7)	6 (5.9)	12 (5.8)
Poor	17 (16.2)	12 (11.9)	29 (14.1)
Neither good nor poor	30 (28.6)	32 (31.7)	62 (30.1)
Good	37 (35.2)	46 (45.5)	83 (40.3)
Very good	15 (14.3)	5 (5.0)	20 (9.7)
AUDIT-3 hazardous alcohol use (n, %) [a]	87 (82.9)	79 (78.2)	166 (80.6)
Any substance use (n, %) [c]	18 (17.1)	19 (18.8)	37 (18.0)
Readiness rulers (M, SD)			
Importance	9.5 (1.2)	9.6 (1.1)	9.5 (1.2)
Readiness	9.1 (1.6)	9.2 (1.7)	9.2 (1.6)
Confidence	4.6 (2.5)	5.0 (2.6)	4.8 (2.5)
BSCQ self-efficacy (M, SD) [a]			
Unpleasant emotions	3.8 (2.8)	4.4 (3.0)	4.1 (2.9)
Physical discomfort	5.4 (3.1)	6.1 (3.0)	5.8 (3.1)
Pleasant emotions	5.6 (3.2)	5.8 (3.0)	5.7 (3.1)
Testing control over gambling	3.8 (2.9)	4.7 (3.0)	4.2 (2.9)
Urges and temptations	3.4 (2.5)	3.9 (2.7)	3.6 (2.6)
Conflict with others	4.5 (3.2)	4.9 (3.1)	4.7 (3.2)
Social pressures to gamble	4.4 (3.4)	4.0 (3.1)	4.2 (3.3)
Having pleasant times with others	5.6 (3.1)	5.3 (3.3)	5.4 (3.2)
Financial pressures [d]	4.0 (3.2)	4.3 (3.4)	4.1 (3.3)
Alcohol or drugs [d]	4.6 (3.6)	5.2 (3.9)	4.9 (3.8)

[a] K6: Kessler 6 Psychological Distress Scale; EUROHIS (first item); AUDIT-3: Alcohol Use Disorders Identification Test-3; BSCQ: Brief Situational Confidence Questionnaire (adapted to gambling)

[b] First item only

[c] Based on previous 30 days

[d] Additional items

Table S3. Baseline sample treatment characteristics

Treatment characteristic	Pure self-directed (n = 105)	Guided self-directed (n = 101)	Total (n = 206)
Treatment goal (n, %)			
Quit altogether	51 (48.6)	49 (48.5)	100 (48.5)
Quit problem gambling activities	25 (23.8)	25 (24.8)	50 (24.3)
Cut back problem gambling activities	29 (27.6)	27 (26.7)	56 (27.2)
HSQ high-intensity help-seeking (n, %) [a, b]			
Gambling counsellor face-to-face	8 (7.6)	4 (4.0)	12 (5.8)
Financial counselling	3 (2.9)	3 (3.0)	15 (7.3)
Residential facility	1 (1.0)	2 (2.0)	3 (1.5)
Gambling support group	3 (2.9)	6 (5.9)	9 (4.4)
Psychologist/psychiatrist/GP	8 (7.6)	7 (6.9)	15 (7.3)
Any high-intensity intervention	15 (14.3)	17 (16.8)	32 (15.5)
HSQ low-intensity help-seeking (n, %) [a, b]			
Gambling helpline	8 (7.6)	6 (5.9)	14 (6.8)
Gambling counsellor online	4 (3.8)	6 (5.9)	10 (4.9)
Gambling counsellor via email	2 (1.9)	1 (1.0)	3 (1.5)
Any low-intensity intervention	12 (11.4)	10 (9.9)	22 (10.7)
HSQ self-directed actions (n, %) [a, b]			
Online gambling forums	12 (11.4)	10 (9.9)	22 (10.7)
Family/friends	32 (30.5)	18 (17.8)	50 (24.3)
Self-help	20 (19.6)	28 (28.0)	48 (23.8)
Read information on Gambling Help Online website	22 (21.0)	24 (23.8)	46 (22.3)
Completed self-help module on website	2 (1.9)	6 (5.9)	8 (3.9)
Self-exclusion	11 (10.5)	5 (5.0)	16 (7.8)
Any self-directed action	53 (50.5)	44 (43.6)	97 (47.1)

[a] HSQ: Help-Seeking Questionnaire
[b] Based on previous 30 days

Table S4. Baseline differences between PSD and GSD participants who completed the 8-week, 12-week or 24-month evaluation

**Socio-demographic characteristic**	**Pure self-directed (n = 38)**	**Guided self-directed (n = 42)**	**p**
Sex (n, %)			
Female	17 (34.0)	18 (35.3)	0.891
Male	33 (66.0)	33 (64.7)	
Age group in years (n, %)			
18 – 24	6 (12.0)	9 (17.7)	0.903
25 – 29	8 (16.0)	6 (11.8)	
30 – 34	9 (18.0)	7 (13.7)	
35 – 39	9 (18.0)	10 (19.6)	
40 – 44	4 (8.0)	4 (7.8)	
45 – 49	2 (4.0)	5 (9.8)	
50 – 54	5 (10.0)	5 (9.8)	
55+	7 (14.0)	5 (9.8)	
Country of birth (n, %)			
Australia	42 (84.0)	39 (76.5)	0.342
Other	8 (16.0)	12 (23.5)	
Employment (n, %)			
Work full-time	36 (72.0)	37 (72.6)	0.508
Work part-time/casual	6 (12.0)	9 (17.7)	
Unemployed	2 (4.0)	1 (2.0)	
Full time student	1 (2.0)	1 (2.0)	
Full-time home duties	0 (0)	1 (2.0)	
Retired	3 (6.0)	0 (0)	
Sick or disability pension	2 (4.0)	1 (2.0)	
Other	0 (0.0)	1 (2.0)	
Annual personal net income (n, %)			
< $25,000	7 (14.0)	5 (9.8)	0.929
$25,000 - $39,999	7 (14.0)	9 (17.7)	
$40,000 - $64,999	14 (28.0)	17 (33.3)	
$65,000 - $79,999	7 (14.0)	8 (15.7)	
$80,000 - $129,999	12 (24.0)	9 (17.7)	
$130,000+	3 (6.0)	3 (5.9)	

Table S5. Patterns of missing data for G-SAS gambling symptom severity

Frequency	Percent (%)	Cumulative	Pattern			
			Baseline	8 weeks	12 weeks	24 months
PSD						
56	53.33	53.33	X			
11	10.48	63.81	X			X
8	7.62	71.43	X	X		
7	6.67	78.10	X		X	
7	6.67	84.76	X		X	X
6	5.71	90.48	X	X	X	
6	5.71	96.19	X	X	X	X
4	3.81	100.00	X	X		X
GSD						
50	49.50	49.50	X			
14	13.86	63.37	X	X	X	X
9	8.91	72.28	X			X
9	8.91	81.19	X		X	
8	7.92	89.11	X	X		
5	4.95	94.06	X	X		X
4	3.96	98.02	X	X	X	
2	1.98	100.00	X		X	X

‘X’ denotes an observed G-SAS gambling symptom severity score

Table S6. Intention-to-treat between-group comparison of PSD and GSD conditions on primary and secondary outcomes [a]

Outcome	Unadjusted estimate (SE)				Estimated between-group difference (95% CI)	p
	Baseline	8-weeks	12-weeks	24-months		
G-SAS gambling symptom severity [b]	-	-0.33 (0.19)	-0.49 (0.29)	-3.94 (2.33)	-0.16 (-0.35, 0.03) [c]	0.091
G-SAS gambling urges [b]	-	-0.19 (0.07)	-0.28 (0.11)	-2.22 (0.85)	-0.09 (-0.16, -0.02) [c]	0.009
Gambling frequency	-	-	-	-	0.95 (0.91, 1.00) [d]	0.043
Gambling expenditure	-	-	-	-	0.99 (0.94, 1.04) [d]	0.728
K6 psychological distress [b]	-	-0.05 (0.10)	-0.08 (0.15)	-0.62 (1.18)	-0.03 (-0.12, 0.07) [c]	0.599
EUROHIS quality of life [b]	-	0.02 (0.02)	0.03 (0.03)	0.26 (0.21)	0.01 (-0.01, 0.03) [c]	0.216
HSQ high-intensity help-seeking [b]	-	-	-	-	0.98 (0.90, 1.05) [e]	0.516
HSQ low-intensity help-seeking [b]	-	-	-	-	0.94 (0.85, 1.04) [e]	0.263

[a] Adjusted for participant age and gender

[b] G-SAS: Gambling Symptom Assessment Scale; K6: Kessler 6 Psychological Distress Scale; EUROHIS (first item); HSQ: Help-Seeking Questionnaire

[c] Linear mixed regression models

[d] Mixed effects ordered logistic regression models - estimates reported as ORs (reference category = PSD)

[e] Mixed effects logistic regression models - estimates reported as ORs (reference category = PSD)

Table S7. Intention-to-treat within-group change for PSD and GSD conditions in primary and secondary outcomes [a]

Outcome	Treatment group	Unadjusted estimate (SE)				Estimated within-group difference (95% CI)	p
		Baseline	8-weeks	12-weeks	24-months		
G-SAS gambling symptom severity [b]	PSD	30.28 (0.89)	21.81 (1.10)	18.09 (1.45)	18.74 (1.79)	-4.58 (-5.71, -3.44) [c]	<0.001
	GSD	28.82 (0.87)	19.70 (1.00)	15.67 (1.32)	14.19 (1.65)	-4.92 (-6.00, -3.85) [c]	<0.001
G-SAS gambling urges [b]	PSD	10.30 (0.33)	7.68 (0.41)	6.53 (0.54)	6.67 (0.62)	-1.41 (-1.83, -1.00) [c]	<0.001
	GSD	9.73 (0.31)	6.92 (0.36)	5.68 (0.48)	4.17 (0.63)	-1.51 (-1.90, -1.12 [c]	<0.001
Gambling frequency	PSD	-	-	-	-	0.67 (0.50, 0.90) [d]	0.007
	GSD	-	-	-	-	0.38 (0.27, 0.54) [d]	<0.001
Gambling expenditure	PSD	-	-	-	-	0.39 (0.26, 0.59) [d]	<0.001
	GSD	-	-	-	-	0.41 (0.30, 0.56) [d]	<0.001
K6 psychological distress [b]	PSD	17.01 (0.52)	14.68 (0.62)	13.65 (0.78)	12.68 (0.82)	-1.26 (-1.80, -0.71) [c]	<0.001
	GSD	17.24 (0.56)	13.91 (0.65)	12.45 (0.84)	12.14 (0.93)	-1.80 (-2.45, -1.15) [c]	<0.001
EUROHIS quality of life [b]	PSD	3.37 (0.10)	3.45 (0.12)	3.49 (0.15)	3.58 (0.17)	0.04 (-0.07, 0.16) [c]	0.451
	GSD	3.32 (0.09)	3.59 (0.11)	3.71 (0.13)	3.83 (0.15)	0.15 (0.04, 0.25) [c]	0.005
HSQ high-intensity help-seeking [b]	PSD	-	-	-	-	1.39 (0.84, 2.31) [e]	0.199
	GSD	-	-	-	-	1.31 (0.84, 2.05) [e]	0.239
HSQ low-intensity help-seeking [b]	PSD	-	-	-	-	1.23 (0.74, 2.06) [e]	0.422
	GSD	-	-	-	-	1.33 (0.87, 2.04) [e]	0.189

[a]

Adjusted for participant age and gender

[b] G-SAS: Gambling Symptom Assessment Scale; K6: Kessler 6 Psychological Distress Scale; EUROHIS (first item); HSQ: Help-Seeking Questionnaire

[c] Linear mixed regression models

[d] Mixed effects ordered logistic regression models - estimates reported as ORs

[e] Mixed effects logistic regression models - estimates reported as ORs

Table S8. Per-protocol between-group comparison of PSD and GSD conditions on primary and secondary outcomes after controlling for help-seeking [a]

Outcome	Unadjusted estimate (SE)				Estimated between-group difference (95% CI)	p
	Baseline	8-weeks	12-weeks	24-months		
G-SAS gambling symptom severity [b]	-	-0.32 (0.19)	-0.49 (0.29)	-3.89 (2.31)	-0.16 (-0.35, 0.03) [c]	0.092
G-SAS gambling urges [b]	-	-0.18 (0.07)	-0.27 (0.11)	-2.19 (0.84)	-0.09 (-0.16, -0.02) [c]	0.010
Gambling frequency	-	-	-	-	0.95 (0.91, 1.00) [d]	0.046
Gambling expenditure	-	-	-	-	0.99 (0.94, 1.04) [d]	0.702
K6 psychological distress [b]	-	-0.06 (0.10)	-0.08 (0.15)	-0.67 (1.18)	-0.03 (-0.12, 0.07) [c]	0.567
EUROHIS quality of life [b]	-	0.02 (0.02)	0.03 (0.03)	0.26 (0.21)	0.01 (-0.01, 0.03) [c]	0.207

[a] Adjusted for participant age, gender, low- and high-intensity help-seeking (Help-Seeking Questionnaire)

[b] G-SAS: Gambling Symptom Assessment Scale; K6: Kessler 6 Psychological Distress Scale; EUROHIS (first item)

[c] Linear mixed regression models

[d] Mixed effects ordered logistic regression models - estimates reported as ORs (reference category = PSD)

Table S9. Per-protocol within-group change for PSD and GSD conditions in primary and secondary outcomes after controlling for help-seeking [a]

Outcome	Treatment group	Unadjusted estimate (SE)				Estimated within-group difference (95% CI)	p
		Baseline	8-weeks	12-weeks	24-months		
G-SAS gambling symptom severity [b]	PSD	29.71 (1.37)	22.10 (1.33)	18.75 (1.60)	18.40 (1.85)	-4.11 (-5.37, -2.85) [c]	<0.001
	GSD	28.09 (1.31)	19.42 (1.20)	15.59 (1.47)	14.03 (1.73)	-4.68 (-5.94, -3.42) [c]	<0.001
G-SAS gambling urges [b]	PSD	10.03 (0.52)	7.60 (0.50)	6.54 (0.61)	6.50 (0.66)	-1.31 (-1.79, -0.83) [c]	<0.001
	GSD	9.15 (0.47)	6.63 (0.42)	5.52 (0.52)	4.01 (0.65)	-1.35 (-1.80, -0.90) [c]	<0.001
Gambling frequency	PSD	-	-	-	-	0.67 (0.49, 0.94) [d]	0.019
	GSD	-	-	-	-	0.36 (0.25, 0.53) [d]	<0.001
Gambling expenditure	PSD	-	-	-	-	0.36 (0.23, 0.56) [d]	<0.001
	GSD	-	-	-	-	0.38 (0.26, 0.55) [d]	<0.001
K6 psychological distress [b]	PSD	16.55 (0.77)	14.39 (0.75)	13.43 (0.86)	12.34 (0.91)	-1.16 (-1.74, -0.58) [c]	<0.001
	GSD	17.28 (0.76)	13.80 (0.70)	12.28 (0.84)	12.25 (0.94)	-1.88 (-2.57, -1.18) [c]	<0.001
EUROHIS quality of life [b]	PSD	3.34 (0.15)	3.44 (0.14)	3.49 (0.17)	3.55 (0.18)	0.05 (-0.07, 0.17) [c]	0.374
	GSD	3.35 (0.13)	3.61 (0.12)	3.73 (0.14)	3.85 (0.16)	0.14 (0.03, 0.25) [c]	0.015

[a] Adjusted for participant age, gender, low- and high-intensity help-seeking (Help-Seeking Questionnaire)

[b] G-SAS: Gambling Symptom Assessment Scale; K6: Kessler 6 Psychological Distress Scale; EUROHIS (first item)

[c] Linear mixed regression models

[d] Mixed effects ordered logistic regression models - estimates reported as ORs

Table S10. Estimated effect of GSD intervention (vs PSD intervention) from per-protocol, multiple imputation under MAR, multiple imputation under LMCF, multiple imputation under J2R, and multiple imputation under CIR

**Analysis**	**8-weeks**		**12-weeks**		**24-months**	
	**Estimate (SE)** [a]	**p**	**Estimate (SE)** [a]	**p**	**Estimate (SE)** [a]	**p**
Per-protocol	1.46 (2.82)	0.606	-2.60 (2.75)	0.359	-1.61 (3.14)	0.614
MAR [b]	2.44 (1.94)	0.216	-2.68 (3.11)	0.416	-1.63 (2.42)	0.516
LMCF [b]	2.44 (1.93)	0.214	0.39 (3.00)	0.899	0.42 (2.05)	0.839
J2R [c]	1.77 (1.74)	0.310	0.11 (2.18)	0.962	0.65 (1.78)	0.718
CIR d	1.77 (1.74)	0.310	0.19 (2.23)	0.932	0.24 (1.72)	0.889

[a] Linear mixed regression models

[b] MAR: randomised-arm missing at random; LMCF: last mean carried forward; J2R: jump to reference; CIR: copy increments in reference

Table S11. Exploratory univariate and multivariate logistic regression models of factors associated with clinically significant change on G-SAS gambling symptom severity at 8- or 12- week evaluation

Variable	Univariate model			Multivariate model [a]		
	OR	95% CI	p	OR	95% CI	p
Sex						
Female (referent)	1.00	-	-	1.00	-	-
Male	0.43	0.16 - 1.14	0.091	1.77	0.26 – 12.1	0.560
Age [b]	1.11	0.90 - 1.37	0.333			
Country of birth						
Australia (referent)	1.00	-	-			
Other	0.77	0.26 -2.29	0.634			
Employment						
Unemployed/student (referent)	1.00	-	-			
Employed full-time/part-time/casual	0.40	0.08 - 2.02	0.266			
Annual personal net income						
< AUD$40,000 (referent)	1.00	-	-	1.00	-	-
AUD$40,000 - $79,999	0.33	0.10 - 1.08	0.067	0.30	0.08 – 1.18	0.086
AUD$80,000+	0.63	0.16 - 2.41	0.500	0.92	0.19 – 4.43	0.915
Internet use (hours) [c]	0.90	0.72 - 1.12	0.349			
Problematic gambling activities						
No EGMs (referent)	1.00	-	-	1.00	-	-
EGMs only	5.50	1.61 - 18.84	0.007	12.83	1.69 – 97.49	0.014
EGMs + others	1.88	0.57 - 6.21	0.304	2.48	0.66 – 9.35	0.179
PGSI problem gambling severity [d]	1.00	0.92 - 1.09	0.912			
Gambling frequency (days) [e]	1.00	0.98 - 1.03	0.778			
Gambling expenditure ($) [e]						
AUD$0 (referent)	1.00	-	-			
AUD$1 - $200	0.67	0.04 - 11.29	0.779			
AUD$201 - $800	4.89	0.68 - 34.96	0.114			
AUD$801+	2.40	0.49 - 11.81	0.282			
K6 psychological distress [d]	1.08	0.99 - 1.17	0.080	1.06	0.97 – 1.17	0.206
EUROHIS quality of life [d,f]	1.07	0.70 - 1.62	0.759			
AUDIT-3 hazardous alcohol use [d]	1.13	0.79 - 1.61	0.509			
Substance use frequency [e]	1.03	0.89 – 1.20	0.679			
Readiness rulers						
Willing	1.81	1.01 - 3.27	0.047	1.79	1.01, 3.17	0.046
Ready	1.38	0.92 - 2.06	0.117			
Able	1.13	0.94 - 1.35	0.190			
BSCQ self-efficacy [d]	1.00	0.98 - 1.03	0.782			
Treatment goal						
Quit altogether (referent)	1.00					
Quit problem gambling activities	0.57	0.20 - 1.67	0.305			
Cut back problem gambling activities	0.51	0.16 - 1.63	0.256			
HSQ high-intensity help-seeking [d, e]						
No (referent)	1.00	-	-			
Yes	2.60	0.77 - 8.78	0.123			
HSQ low-intensity help-seeking [d, e]						
No (referent)	1.00	-	-			
Yes	3.29	0.67 - 16.21	0.143			
HSQ self-directed actions [d, e]						
No (referent)	1.00	-	-			
Yes	1.57	0.62 - 3.96	0.337			
Treatment engagement [g]	1.02	0.98 - 1.06	0.338			

[a] Variable selection based on $p < 0.10$ from univariable analyses

[b] Age groups in continuous form

[c] Based on average weekly use for work/personal/education/recreation

[d] PGSI: Problem Gambling Severity Index; K6: Kessler 6 Psychological Distress Scale; EUROHIS (first item); AUDIT-3: Alcohol Use Disorders Identification Test-3; BSCQ: Brief Situational Confidence Questionnaire (adapted to gambling); HSQ: Help-Seeking Questionnaire

[e] Based on previous 30 days

[f] First item only

[g] Treatment engagement defined as completing at least one module activity

Table S12. Exploratory univariate and multivariate logistic regression models of factors associated with clinically significant change on G-SAS gambling symptom severity at 24-month evaluation

Variable	Univariate model			Multivariate model [a]		
	OR	95% CI	p	OR	95% CI	p
Sex						
Female (referent)	1.00	-	-	1.00	-	-
Male	0.27	0.07 – 1.09	0.065	0.19	0.04 – 0.85	0.030
Age [b]	0.98	0.75 – 1.26	0.859			
Country of birth						
Australia (referent)	1.00	-	-			
Other	1.67	0.40 – 6.97	0.484			
Employment						
Unemployed/student (referent)	1.00	-	-			
Employed full-time/part-time/casual	1.80	0.36 – 9.04	0.475			
Annual personal net income						
< AUD$40,000 (referent)	1.00	-	-			
AUD$40,000 - $79,999	0.96	0.21 – 4.45	0.957			
AUD$80,000+	0.38	0.07 – 1.99	0.253			
Internet use (hours) [c]	1.45	1.05 – 2.01	0.025	1.59	1.09 – 2.30	0.015
Problematic gambling activities						
No EGMs (referent)	1.00	-	-			
EGMs only	2.92	0.65 –	0.160			
EGMs + others	1.55	0.34 – 6.94	0.568			
PGSI problem gambling severity [d]	1.00	0.91 – 1.09	0.978			
Gambling frequency (days) [e]	1.00	0.97 – 1.03	0.839			
Gambling expenditure ($) [e]						
AUD$0 (referent)	1.00	-	-			
AUD$1 - $200	1.00	-	-			
AUD$201 - $800	0.38	0.02 – 6.35	0.497			
AUD$801+	0.53	0.05 – 5.19	0.588			
K6 psychological distress [d]	1.07	0.97 – 1.17	0.172			
EUROHIS quality of life [d,f]	1.10	0.66 – 1.82	0.717			
AUDIT-3 hazardous alcohol use [d]	0.72	0.46 – 1.13	0.148			
Substance use frequency [e]	1.07	0.87 – 1.32	0.517			
Readiness rulers						
Willing	0.75	0.43 – 1.29	0.298			
Ready	0.98	0.76 – 1.25	0.852			
Able	1.05	0.84 – 1.32	0.673			
BSCQ self-efficacy [d]	1.00	0.97 – 1.02	0.753			
Treatment goal						
Quit altogether (referent)	1.00	-	-			
Quit problem gambling activities	2.65	0.61 – 11.4	0.192			
Cut back problem gambling activities	1.38	0.34 – 5.56	0.655			
HSQ high-intensity help-seeking [d, e]						
No (referent)	1.00	-	-			
Yes	1.02	0.27 – 3.87	0.981			
HSQ low-intensity help-seeking [d, e]						
No (referent)	1.00	-	-			
Yes	1.14	0.20 – 6.53	0.881			
HSQ self-directed actions [d, e]						
No (referent)	1.00					
Yes	1.13	0.37 – 3.46	0.829			
Treatment engagement [g]	1.04	0.97 – 1.11	0.251			

[a] Variable selection based on p < 0.10 from univariable analyses

[b] Age groups in continuous form

[c] Based on average weekly use for work/personal/education/recreation

[d] PGSI: Problem Gambling Severity Index; K6: Kessler 6 Psychological Distress Scale; EUROHIS (first item); AUDIT-3: Alcohol Use Disorders Identification Test-3; BSCQ: Brief Situational Confidence Questionnaire (adapted to gambling); HSQ: Help-Seeking Questionnaire

[e] Based on previous 30 days

[f] First item only

[g] Treatment engagement defined as completing at least one module activity

Table S13. Exploratory univariate and multivariate logistic regression models of factors associated with module activity completion

Variable	Univariate model OR	95% CI	p	Multivariate model [a] OR	95% CI	p
Sex						
Female (referent)	1.00	-	-			
Male	0.63	0.35 – 1.15	0.130			
Age [b]	1.24	1.09 – 1.41	0.001	1.28	1.11 – 1.47	0.001
Country of birth						
Australia (referent)	1.00	-	-			
Other	0.76	0.38 – 1.54	0.450			
Employment						
Unemployed/student (referent)	1.00	-	-			
Employed full-time/part-time/casual	1.55	0.59 – 4.10	0.378			
Annual personal net income						
< AUD$40,000 (referent)	1.00	-	-			
AUD$40,000 - $79,999	1.20	0.60 – 2.41	0.611			
AUD$80,000+	1.01	0.43 – 2.37	0.983			
Internet use (hours) [c]	1.20	1.04 – 1.39	0.013	1.29	1.10 – 1.52	0.002
Problematic gambling activities						
No EGMs (referent)	1.00	-	-			
EGMs only	1.82	0.86 – 3.85	0.116			
EGMs + others	1.19	0.53 – 2.65	0.678			
PGSI problem gambling severity [d]	0.96	0.91 – 1.01	0.151			
G-SAS gambling symptom severity [d]	0.99	0.95 – 1.03	0.532			
G-SAS gambling urges [d]	0.95	0.85 – 1.05	0.296			
Gambling frequency (days) [e]	0.99	0.97 – 1.01	0.426			
Gambling expenditure ($) [e]						
AUD$0 (referent)	1.00	-	-			
AUD$1 - $200	0.83	0.19 – 3.58	0.806			
AUD$201 - $800	2.34	0.75 – 7.37	0.145			
AUD$801+	0.75	0.30 – 1.91	0.552			
K6 psychological distress [d]	0.98	0.93 – 1.03	0.353			
EUROHIS quality of life [d,f]	1.21	0.90 – 1.62	0.200			
AUDIT-3 hazardous alcohol use [d]	0.83	0.65 – 1.06	0.145			
Substance use frequency [e]	0.92	0.83 – 1.02	0.124			
Readiness rulers						
Willing	0.96	0.75 – 1.22	0.737			
Ready	1.05	0.87 – 1.26	0.622			
Able	1.01	0.90 – 1.13	0.929			
BSCQ self-efficacy [d]	1.01	1.00 – 1.03	0.089	1.02	0.00 – 1.03	0.031
Treatment goal						
Quit altogether (referent)	1.00	-	-			
Quit problem gambling activities	1.67	0.83 – 3.36	0.151			
Cut back problem gambling activities	0.71	0.34 - 1.48	0.359			
HSQ high-intensity help-seeking [d, e]						
No (referent)	1.00	-	-			
Yes	1.26	0.58 – 2.77	0.557			
HSQ low-intensity help-seeking [d, e]						
No (referent)	1.00	-	-			
Yes	0.94	0.36 – 2.43	0.900			
HSQ self-directed actions [d, e]						
No (referent)	1.00	-	-	1.00	-	-
Yes	2.68	1.47 – 4.87	0.001	2.93	1.54 – 5.60	0.001

[a] Variable selection based on $p < 0.10$ from univariable analyses

[b] Age groups in continuous form

[c] Based on average weekly use for work/personal/education/recreation

[d] PGSI: Problem Gambling Severity Index; G-SAS: Gambling Symptom Assessment Scale; K6: Kessler 6 Psychological Distress Scale; EUROHIS (first item); AUDIT-3: Alcohol Use Disorders Identification Test-3; BSCQ: Brief Situational Confidence Questionnaire (adapted to gambling); HSQ: Help-Seeking Questionnaire

[e] Based on previous 30 days

[f] First item only

Table S14. Exploratory univariate and multivariate logistic regression models of factors associated with post-baseline evaluation completion

Variable	Univariate model			Multivariate model [a]		
	OR	95% CI	p	OR	95% CI	p
Sex						
Female (referent)	1.00	-	-			
Male	1.07	0.60 – 1.89	0.818			
Age [b]	1.10	0.98 – 1.24	0.103			
Country of birth						
Australia (referent)	1.00	-	-			
Other	0.65	0.34 – 1.24	0.189			
Employment						
Unemployed/student (referent)	1.00	-	-			
Employed full-time/part-time/casual	0.79	0.34 – 1.86	0.593			
Annual personal net income						
< AUD$40,000 (referent)	1.00	-	-			
AUD$40,000 - $79,999	0.74	0.38 – 1.42	0.365			
AUD$80,000+	1.45	0.65 – 3.21	0.364			
Internet use (hours) [c]	1.13	0.98 – 1.30	0.087	1.11	0.95 – 1.29	0.199
Problematic gambling activities						
No EGMs (referent)	1.00	-	-			
EGMs only	0.92	0.46 – 1.82	0.809			
EGMs + others	0.88	0.43 – 1.81	0.729			
PGSI problem gambling severity [d]	1.01	0.96 – 1.07	0.632			
G-SAS gambling symptom severity [d]	0.98	0.94 – 1.01	0.227			
G-SAS gambling urges [d]	0.90	0.81 – 0.99	0.034	0.90	0.80 – 1.00	0.047
Gambling frequency (days) [e]	1.00	0.99 – 1.02	0.619			
Gambling expenditure ($) [e]						
AUD$0 (referent)	1.00	-	-	1.00	-	-
AUD$1 - $200	0.83	0.19 – 3.58	0.806	1.17	0.24 – 5.66	0.845
AUD$201 - $800	3.19	1.00 –	0.050	3.64	1.00 – 13.27	0.051
AUD$801+	1.90	0.76 – 4.76	0.170	2.78	0.97 – 7.98	0.058
K6 psychological distress [d]	0.99	0.94 – 1.04	0.634			
EUROHIS quality of life [d,f]	0.99	0.76 – 1.30	0.965			
AUDIT-3 hazardous alcohol use [d]	0.90	0.72 – 1.13	0.378			
Substance use frequency [e]	0.96	0.90 – 1.02	0.198			
Readiness rulers						
Willing	1.01	0.80 – 1.27	0.951			
Ready	0.87	0.73 – 1.04	0.121			
Able	1.00	0.90 – 1.11	0.996			
BSCQ self-efficacy [d]	1.01	0.99 – 1.02	0.255			
Treatment goal						
Quit altogether (referent)	1.00	-	-			
Quit problem gambling activities	1.23	0.57 – 2.23	0.729			
Cut back problem gambling activities	0.67	0.35 – 1.30	0.235			
HSQ high-intensity help-seeking [d, e]						
No (referent)	1.00	-	-	1.00	-	-
Yes	2.65	1.18 - 5.92	0.018	2.93	1.23 – 6.97	0.015
HSQ low-intensity help-seeking [d, e]						
No (referent)	1.00	-	-			
Yes	1.58	0.64 – 3.87	0.321			
HSQ self-directed actions [d, e]						
No (referent)	1.00	-	-			
Yes	1.53	0.88 – 2.65	0.129			
Treatment engagement [g]	1.07	1.02 – 1.12	0.003	1.06	1.01 – 1.11	0.010

[a] Variable selection based on p < 0.10 from univariable analyses

[b] Age groups in continuous form

[c] Based on average weekly use for work/personal/education/recreation

[d] PGSI: Problem Gambling Severity Index; G-SAS: Gambling Symptom Assessment Scale; K6: Kessler 6 Psychological Distress Scale; EUROHIS (first item); AUDIT-3: Alcohol Use Disorders Identification Test-3; BSCQ: Brief Situational Confidence Questionnaire (adapted to gambling); HSQ: Help-Seeking Questionnaire

[e] Based on previous 30 days

[f] First item only

[g] Treatment engagement defined as completing at least one module activity
